# Comprehensive genomic characterization of hematologic malignancies at a pediatric tertiary care center

**DOI:** 10.3389/fonc.2024.1498409

**Published:** 2024-12-02

**Authors:** Ann M. Kebede, Elizabeth A. R. Garfinkle, Mariam T. Mathew, Elizabeth Varga, Susan I. Colace, Gregory Wheeler, Benjamin J. Kelly, Kathleen M. Schieffer, Katherine E. Miller, Elaine R. Mardis, Catherine E. Cottrell, Samara L. Potter

**Affiliations:** ^1^ Division of Pediatric Heme/Onc/BMT, Nationwide Children’s Hospital, Columbus, OH, United States; ^2^ The Steve and Cindy Rasmussen Institute for Genomic Medicine, Nationwide Children’s Hospital, Columbus, OH, United States; ^3^ Department of Pathology and Laboratory Medicine, The Ohio State University, Columbus, OH, United States; ^4^ Department of Pediatrics, The Ohio State University, Columbus, OH, United States

**Keywords:** genomics, precision medicine, next generation sequencing, therapeutic targets, hematologic malignancies, pediatric oncology

## Abstract

Despite the increasing availability of comprehensive next generation sequencing (NGS), its role in characterizing pediatric hematologic malignancies remains undefined. We describe findings from comprehensive genomic profiling of hematologic malignancies at a pediatric tertiary care center. Patients enrolled on a translational research protocol to aid in cancer diagnosis, prognostication, treatment, and detection of cancer predisposition. Disease-involved samples underwent exome and RNA sequencing and analysis for single nucleotide variation, insertion/deletions, copy number alteration, structural variation, fusions, and gene expression. Twenty-eight patients with hematologic malignancies were nominated between 2018-2021. Eighteen individuals received both germline and somatic sequencing; two received germline sequencing only. Germline testing identified patients with cancer predisposition syndromes and non-cancer carrier states. Fifteen patients (15/18, 83%) had cancer-relevant somatic findings. Potential therapeutic targets were identified in seven patients (7/18, 38.9%); three (3/7, 42.9%) received targeted therapies and remain in remission an average of 47 months later.

## Introduction

Hematologic malignancies, particularly acute leukemias, remain the most commonly diagnosed pediatric malignancies (1–3). Significant improvements have been made in the treatment of pediatric hematologic malignancies since the 1960s, especially in acute lymphoblastic leukemia where survival rates now exceed 80-90% (4, 5). Improved outcomes for pediatric patients with leukemias and lymphomas can be attributed to several factors, including current risk stratification, conventional chemotherapeutic approaches, and improved supportive care, leading to what is considered standard of care today (3–6).

Additionally, research has established that recurrent genomic variants play a role in cancer development, including hematologic malignancies (3, 7–12). In some cases, these alterations have defined disease subtypes, thereby impacting clinical risk stratification or prognosis for pediatric leukemia and lymphoma patients (1, 3, 10, 13). The advent of next generation sequencing (NGS) combined with large-scale consortium-based clinical trials has accelerated the identification of these alterations, such that patients with pediatric leukemia and lymphoma now receive more personalized care, with improved risk stratification and increasing use of targeted agents (14–16). However, most clinical sequencing in this patient population is achieved through panel testing, due in large part to cost and reimbursement constraints. Unfortunately, panel testing, which sequences only a subset of genes and hotspots, may not identify all potential clinically applicable genomic variants including prognostic copy number alterations.

While strides have been made in the survival of pediatric hematologic malignancies, some patient populations remain difficult to treat (6, 12, 17–19). These high-risk patients may ultimately require therapy that is not considered standard of care due to disease progression, therapeutic toxicity, or genetic comorbidities. Comprehensive genomic characterization of both germline and somatic disease tissues can be especially impactful in such high-risk patients. Increasingly expansive NGS technologies can discover genomic alterations that might otherwise go undetected through standard testing approaches, namely cytogenetic studies and panel-based testing. Such findings can have important diagnostic and prognostic implications for patients. Furthermore, broad genomic profiling can suggest potential therapeutic targets for those particularly difficult to treat malignancies, which may improve outcomes (7, 8, 20–23). This knowledge could strengthen our understanding of genetic drivers of high-risk disease as well as prompt further study in clinical trials and disease interventions.

Here we report the results of 18 pediatric and adolescent/young adult (AYA) individuals diagnosed with hematologic malignancies who underwent comprehensive genomic profiling as part of this translational research protocol at a single tertiary care pediatric institution. To evaluate the clinical utility of genomic profiling for this clinician-nominated pediatric/AYA population, paired exome sequencing analysis for the detection of germline and somatic variation, as well as RNA sequencing of disease-involved samples was performed. The goal of this study was to identify genomic alterations of diagnostic, prognostic, and therapeutic impact. Notably, this cohort was largely comprised of patients with relapsed or high-risk disease. We also detail outcomes of those patients who received targeted therapy based upon sequencing results.

## Methods

### Patient recruitment and overview of translational study protocol

Nationwide Children’s Hospital Institutional Research Ethics Board approved this study [IRB17-00206]. Clinical providers nominated patients for enrollment on to the Institute for Genomic Medicine Comprehensive Profiling for Cancer, Blood, and Somatic Disorders protocol. Once patients were approved for the study and informed consent was obtained, testing on available disease-involved and germline samples was initiated. Results were communicated to clinical providers and discussed within multi-disciplinary tumor boards. Upon provider request, results were also clinically confirmed using orthogonal testing. Genetic counseling and cascade testing were offered to enrolled patients as a part of this study.

### Germline and somatic exome sequencing

Libraries were prepared using 100-500ng of input DNA beginning with enzymatic fragmentation, followed by end repair, 5’ phosphorylation, A-tailing, and platform-specific adapter ligation using NEB Ultra II FS reagents (New England Biolabs, Ipswich, MA). Target enrichment by hybrid capture was performed using the IDT xGen Exome Research Panel v1.0 enhanced with the xGenCNV Backbone and Cancer-Enriched Panels-Tech Access (Integrated DNA Technologies, Coralville, IA). Paired-end 151-bp reads were generated on the Illumina HiSeq 4000 or NovaSeq 6000 (Illumina, San Diego, CA). Secondary analysis was performed using Churchill, a comprehensive workflow for analysis of raw reads from genome alignment through to germline and somatic variant identification (112). Reads were aligned to the human genome reference sequence (build GRCh38) using BWA (v0.7.15) (RRID : SCR_010910) and refined according to community-accepted guidelines for best practices (https://gatk.broadinstitute.org/hc/en-us). Duplicate sequence reads were removed using samblaster-v.0.1.22, and base quality score recalibration was performed on the aligned sequence data using the Genome Analysis Toolkit (v4.1.9) (RRID : SCR_001876) (113). Germline variants were called using GATK’s HaplotypeCaller (RRID : SCR_001876) (114), somatic single nucleotide variation (SNV) and insertion-deletion (indel) detection was performed using MuTect2 (RRID : SCR_000559) (115) and copy number variation (CNV) was assessed using a combination of GATK (v4.2.4.1) (RRID : SCR_001876) and VarScan2 (v2.4.4) (RRID : SCR_006849) (116). Germline and somatic variation in cancer-associated genes was assessed using a gene-set curated from the published literature and genomic databases including those described by Zhang et al., as well as genes with strong or emerging evidence of germline or somatic cancer association as documented in the Cancer Gene Census (117, 118).

### RNA-sequencing

Extracted RNA from tumor tissue was subjected to DNase treatment and ribodepletion. For library preparation, the NEBNext Ultra II Directional RNA library prep kit for Illumina was employed. Paired-end 151-bp reads were then generated from the tumor RNA libraries using the Illumina HiSeq 4000 and aligned to the human genome reference sequence (GRCh38) using STAR-Fusion. For fusion analysis, RNA sequence data were processed using EnFusion (119), an ensemble approach that merges and normalizes the results from seven fusion callers: STARfusion (v.1.6.0) (119, 120), MapSplice (v.2.2.1) (121), FusionCatcher (v.0.99.7c) (122), FusionMap (v.mono-2.10.9) (123), JAFFA (v.1.09) (124), CICERO (v0.3.0) (125), and Arriba (v1.2.0) (126). Rare fusions (<5% frequency in our internal cohort) identified by at least three fusion callers were subject to further review for biological relevance. For gene expression studies, normalized read counts (TPM) were calculated for all samples from an internal NCH cohort of CNS cancers (n=508) using Salmon (v.1.9.0). An external cohort of CNS cancers (n=791) from the Treehouse Childhood Cancer Initiative at the UC Santa Cruz Genomics Institute (v.9 and v.11, University of California, Santa Cruz) was combined with the internal cohort to extend the dataset. Expression counts of protein-coding genes were log2(x+1) transformed and quantile normalization was performed. The 5,000 genes with the highest variances were used to perform a principal components analysis.

### Graphical representation of data

Sankey plot was generated using the ggsankey package in R version 4.1.1. Bar graph and pie charts were generated using ggplot2 (RRID : SCR_014601) in R version 4.1.1. OncoPrint was generated using ComplexHeatmap (RRID : SCR_017270) in R version 4.1.1. Swimmer plot was generated using the swimplot package in R version 4.1.1. Code is available upon request.

## Results

### Cohort

**Figure 1:**
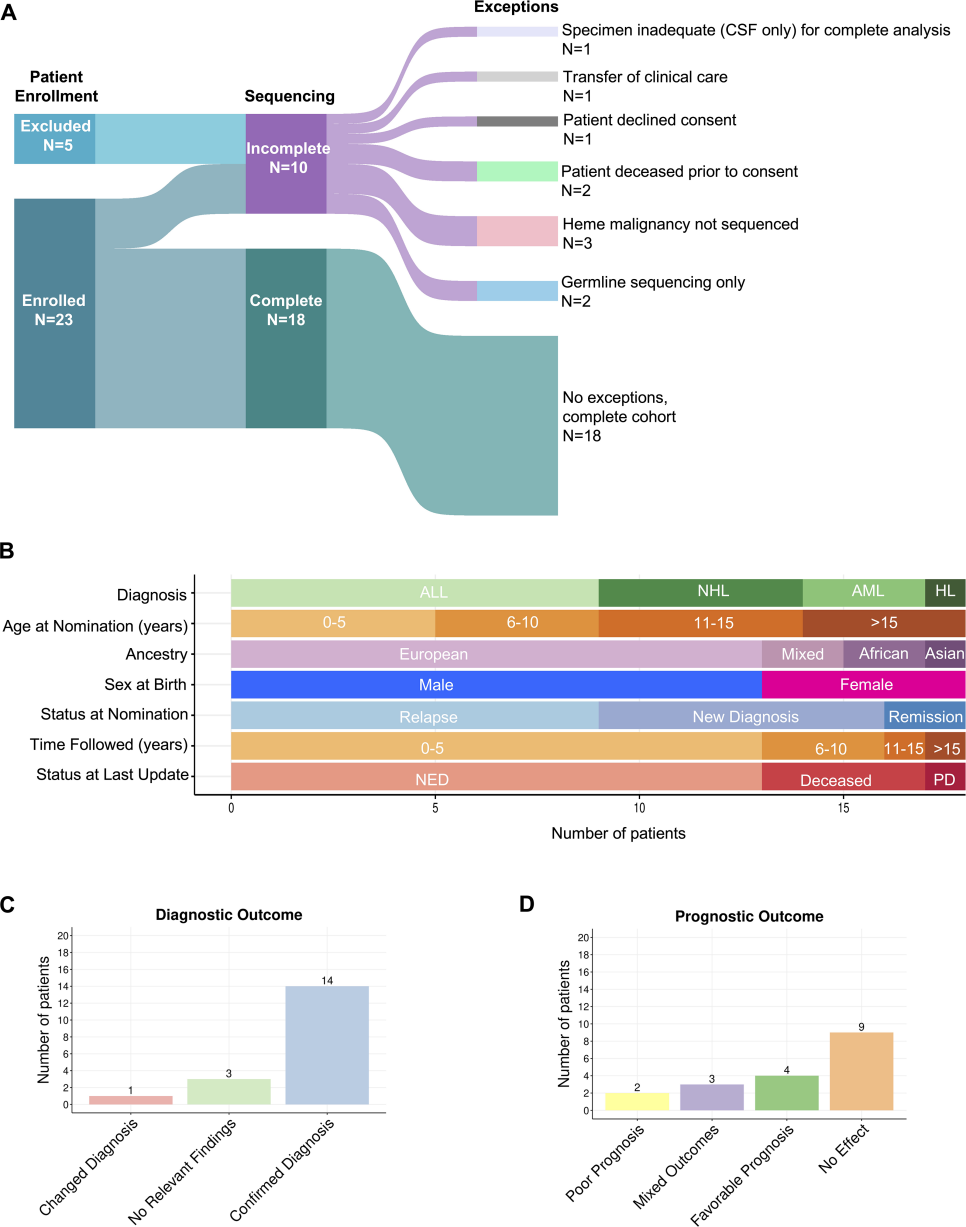
Overview of cohort. **(A)** Sankey plot displaying number of patients that were enrolled and sequenced. Lines flowing between columns represent the relationship between the blocks and their width corresponds to the number of patients. **(B)** Graphical representation of cohort demographics. **(C)** Number of patients and their diagnostic impact from protocol enrollment. **(D)** Number of patients and their prognostic impact from protocol enrollment.

Twenty-eight patients with hematologic malignancies were nominated by clinicians; 23 of these were enrolled (**Figure 1A**). Eighteen patients underwent paired somatic disease-germline comparator exome sequencing and RNA sequencing of a disease-involved sample. Germline only exome sequencing was performed for two additional patients, both of whom were enrolled following remission of their hematologic malignancies, and for whom other, non-hematologic tumor samples were sequenced as a part of this protocol. Five patients were excluded after nomination due to inadequate specimen, decline of consent onto the study, death prior to enrollment, transfer of clinical care, or sequencing not completed on their hematologic malignancy. Sequencing was not completed on the hematologic malignancy of one patient due to lack of a disease-involved sample, and two patients with prior history of hematologic malignancy had only non-hematologic malignancies sequenced (retinoblastoma and osteosarcoma in one individual and malignant peripheral nerve sheath tumor in the other individual). As summarized in **Figure 1B**, diagnoses included acute lymphoblastic leukemia, acute myeloid leukemia, Hodgkin lymphoma, and non-Hodgkin lymphoma. Most patients were male (72.2% vs 27.8%) and ranged in age from 0 to 26 years, with the majority being younger than 15 years old (**Supplementary Table 1**).

Rationale for patient nomination is detailed in **Supplementary Table 2**. The presence of relapsed or refractory disease was the most common reason for clinician nomination (9/18, 50%). Two individuals (2/18, 11.1%) were nominated due to having multiple malignancies; one with concurrent Burkitt Lymphoma and Neuroblastoma at initial diagnosis, the other with a history of Hodgkin Lymphoma at initial diagnosis later followed by Ewing Sarcoma/PNET. Clinicians sought potential therapeutic options for individuals with poor prognoses in two others; one with T-ALL and CD4/CD8 double negative immunophenotypeand one with infantile B-ALL (2/18, 11.1%). Clinicians nominated another two patients due to uncertainty of diagnosis. One patient was negative for c-myc on fluorescence *in situ* hybridization (FISH) analysis, and another had insufficient sample to confirm diagnosis by routine methods (2/18, 11.1%). Individuals were also nominated due to unusual neurologic symptoms (1/18, 5.6%), unusual clinical presentation (1/18, 5.6%), and significant therapeutic toxicity (1/18, 5.6%).

Clinical utility of testing was evaluated based on diagnostic, prognostic, and therapeutic impact. Diagnostic variants were categorized by effect on prior histologic diagnosis, wherein variants were found to confirm, change, or have no effect on histologic diagnosis. Amid genomic characterization that led to a changed histologic diagnosis, these variants were sub-categorized into those that challenged a prior histologic diagnosis, and those that refined a pathologic diagnosis (**Supplementary Table 3**). Genomic characterization led to confirmation of diagnosis for the majority of the cohort (14/18, 77.8%) (7, 24–85). One patient (1/18, 5.5%) had a fusion that refined diagnosis from anaplastic large cell lymphoma (ALCL) to ALK-fusion positive ALCL (IGMCH0009, **Figure 1C**, **Supplementary Table 4**) (58–60). No relevant diagnostic findings were found in three patients (3/18, 16.7%) (**Figure 1C**). For one patient (IGMCH0423), a translocation involving chromosomes 8 and 22 (t(8;22)(q24.2;q11.2)) involving the *MYC* and *IGL* locus, recurrently observed in Burkitt lymphoma, was identified on standard of care karyotyping (**Supplementary Table 4**). The expected resultant rearrangement, however, was not detected by RNA-sequencing, likely due to the nature of the genomic breakpoints. Single nucleotide variants (SNVs) were the most identified diagnostic alterations, followed by fusions, and then copy number variants (CNVs). Less common were insertions/deletions (indels) and internal tandem duplication (ITD).

Prognostic variants were categorized as conferring favorable prognosis, poor prognosis, mixed/intermediate prognosis, and no effect (**Supplementary Table 5**; **Figure 1D**). Additionally, each patient was denoted as having an overall clinical prognostic category (i.e. favorable, poor, or mixed) based on a cumulative assessment of their combined variants. Variants conveying favorable prognosis were identified in 22.2% of the cohort (4/18), including *NOTCH1 SNVs, NPM1::ALK* fusion, and copy number gains (52–54, 75, 77, 86, 87). The mixed clinical prognosis category included patients with multiple variants conferring different prognostic implications (i.e. favorable, poor, intermediate), and variants that have conflicting evidence for prognostication. Three patients (3/18, 16.7%) had variants that resulted in mixed prognosis (35, 43, 45, 46, 51–54, 64, 65, 86–92). Two patients were noted to have overall poor clinical prognosis based on their cumulative variants. (2/18, 11.1%). In one patient with B-ALL, this included an SNV in *NT5C2* and a *KMT2A* fusion. In another patient with myeloid sarcoma and history of treatment-related AML, this included an *ASXL1* indel and *FLT3* ITD (52, 53, 82–87, 93–96). Nine patients did not have genomic findings that affected prognosis (9/18, 50%).

**Figure 2:**
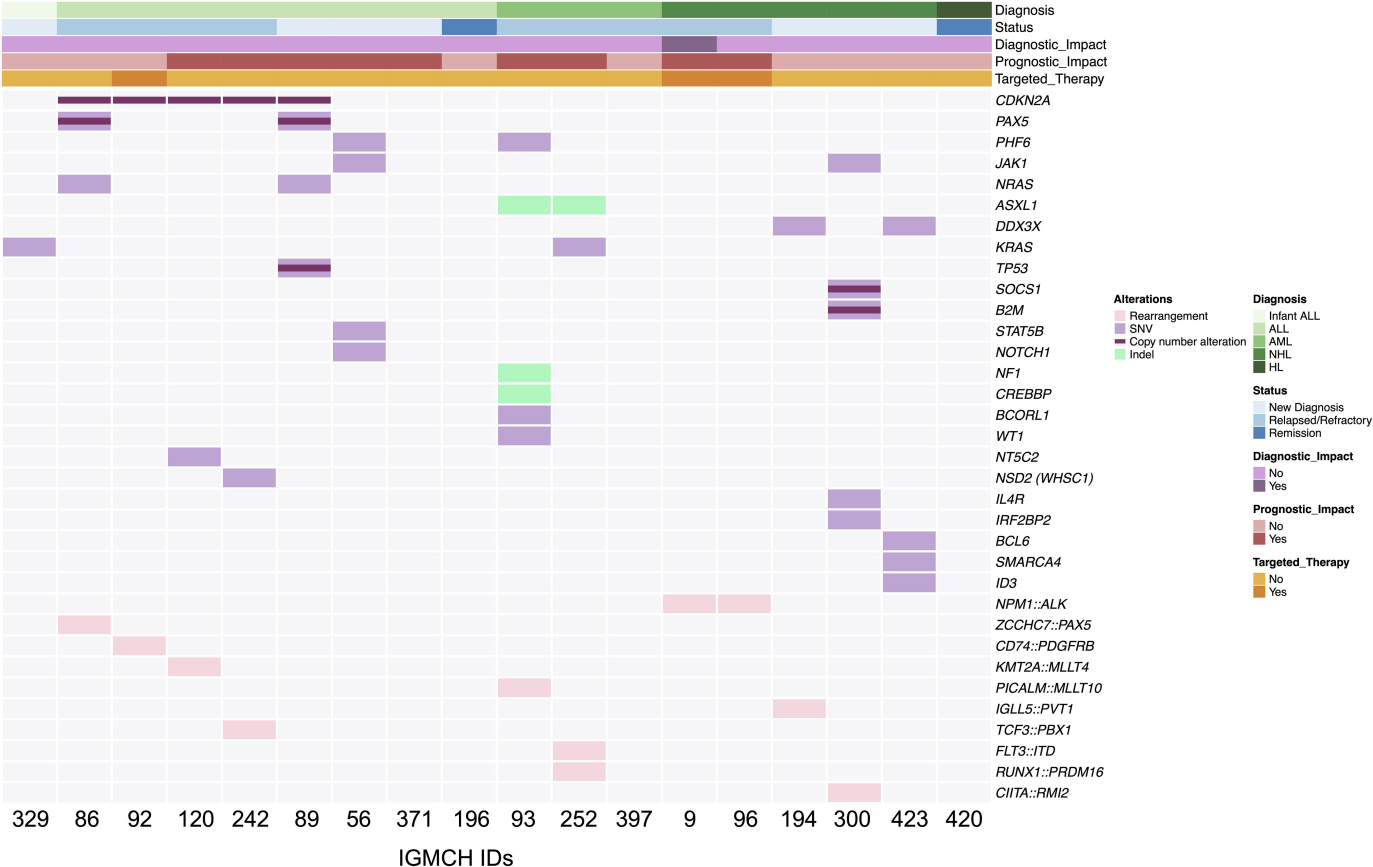
Summary of somatic findings. OncoPrint displaying an overview of somatic genetic alterations. Each column represents an individual patient (n=18) ordered by diagnosis. Status at nomination, diagnostic and prognostic impact from cohort enrollment, and use of targeted therapy are notated with annotation bars. Rearrangements, single nucleotide variants (SNV), copy number alterations, and insertions/deletions (Indel) are represented by a colored line or box. Light grey boxes represent absence of genetic alteration at that particular gene. ALL, acute lymphoblastic leukemia; AML, acute myeloid leukemia; NHL, non-Hodgkin lymphoma; HL, Hodgkin lymphoma.

Sequencing identified 61 somatic variants in 15 patients. Of these patients, seven were newly diagnosed with malignancy, whereas eight patients had relapsed/refractory disease. Six of the seven (85.7%) newly diagnosed patients were advanced stage (Stage III or IV) or high-risk based on age or diagnostic features. Variations in *CDKN2A* were most frequent (5 variants) (**Figure 2**).

**Figure 3:**
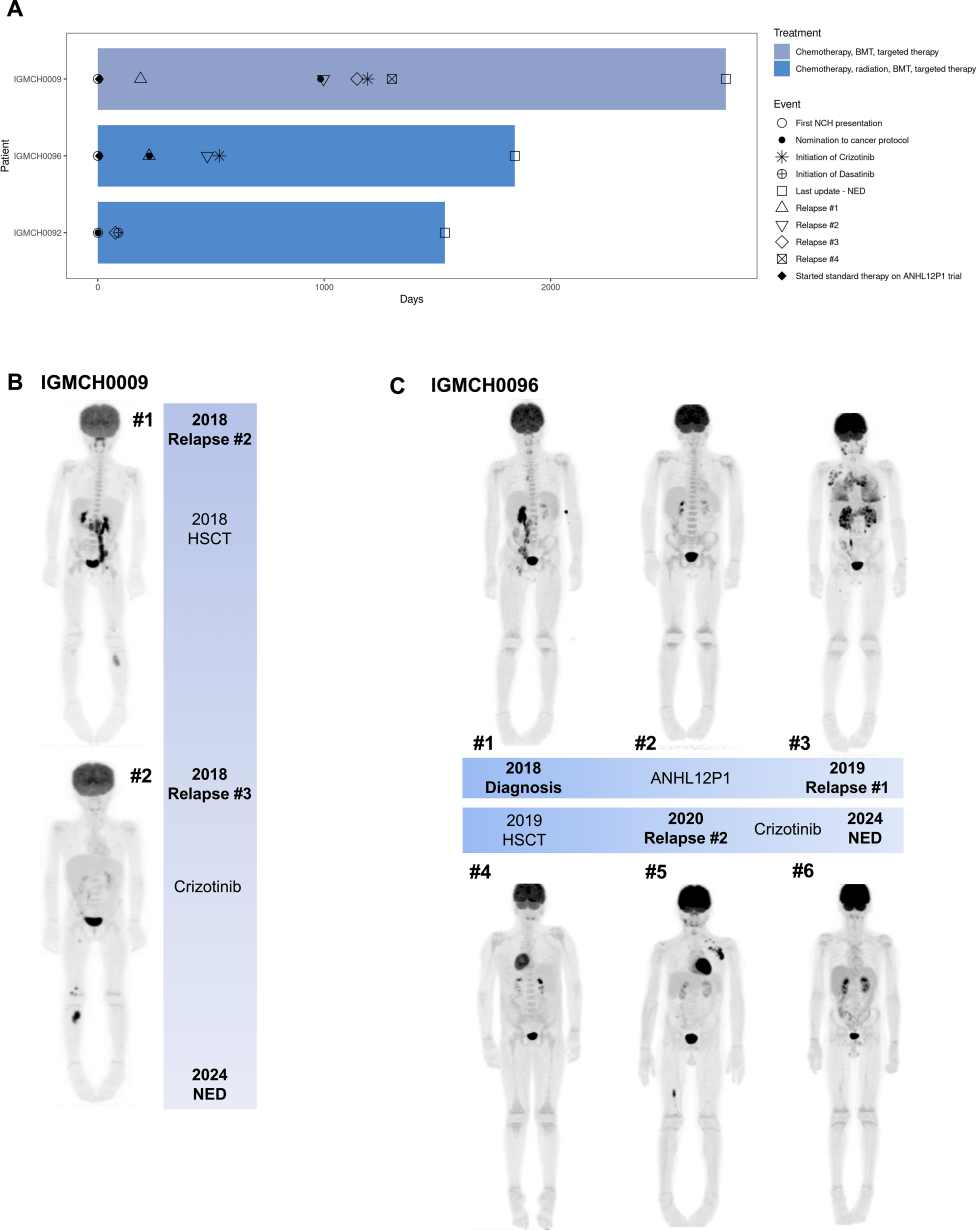
Patients treated with targeted therapy after protocol enrollment. **(A)** Swimmer plot of clinical course of treatment and outcomes for three patients who received targeted therapy after protocol enrollment. Patient IGMCH0009 and Patient IGMCH0096 were diagnosed with anaplastic large-cell lymphoma (ALCL) and received crizotinib targeted therapy. Patient IGMCH0092 was diagnosed with B-cell acute lymphoblastic leukemia (B-ALL) and received dasatinib targeted therapy. Patient IGMCH0092 was enrolled on the protocol at the time of their second relapse. Days = days from first presentation at Nationwide Children’s Hospital. BMT = bone marrow transplant. NED = no evidence of disease. **(B)** PET scans for Patient IGMCH0009 at #1 second relapse and #2 third relapse. **(C)** PET scans for Patient IGMCH0096 throughout course of therapy. #1 at diagnosis, #2 after initial standard of care therapy, #3 first relapse, #4 after hematopoietic stem cell transplant (HSCT), #5 second relapse, #6 after starting crizotinib.

Seven patients had at least one therapeutic target identified (7/18, 38.9%), and to date, three (3/7, 42.9%) of those patients received targeted therapy based upon these results (28, 40, 41, 58–60, 85, 97) (**Table 1**). Variants identified as targets included fusions, SNVs, and CNVs. The three patients who received targeted therapy had no evidence of disease at last update, an average of 47 months’ duration (**Figure 3A**).

**Table 1:** Therapeutic findings.

Patient (IGMCH)	Diagnosis	Somatic Variant	Potential Targeted Agent Identified	Treatment	Outcome
**0009**	ALCL	*NPM1::ALK* (NM_002520, NM_004304) (58–60)	ALK-inhibitor (Crizotinib)	Chemotherapy BMT Targeted Therapy	NED
**0086**	B-ALL	*NRAS* c.183A>T (p.Gln61His) (NM_002524.4) (28, 97)	MEK- inhibitor (Trametinib)	Chemotherapy	NED
**0089**	B-ALL	*NRAS* c.35G>A (p.Gly12Asp) (NM_002524) (28, 97)	MEK- inhibitor (Selumetinib)	Chemotherapy	NED
**0092**	B-ALL	*CD74::PDGFRB (* 41, 107)	Tyrosine Kinase- Inhibitor (Dasatinib)	Chemotherapy Radiation BMT Targeted Therapy	NED
**0096**	ALCL	*NPM1::ALK* (NM_002520.6, NM_004304.5) (58–60)	ALK- inhibitor (Crizotinib)	Chemotherapy Radiation Targeted Therapy	NED
**0252**	AML	*KRAS* c.37G>T (p.Gly13Cys) (NM_033360.4>NM_033360.3) (28, 97) *FLT3*-ITD (M_004119.3>NM_004119.2) (85)	MEK- inhibitor (Trametinib) *FLT3*-ITD (Gilteritinib)	Chemotherapy Radiation Surgery BMT	Relapse
**0300**	PMBCL	*JAK1* c.1872A>T (p.Leu624Phe) (NM_002227.4) (40) Chr 9 CNV (gain) (40)	PD-L1- inhibitor (Pembrolizumab)	Chemotherapy	NED

ALCL, anaplastic large cell lymphoma; B-ALL, B-cell acute lymphoblastic leukemia; AML, acute myeloid leukemia; PMBCL - primary mediastinal B cell lymphoma; CNV, copy number variant; ITD, internal tandem duplication; BMT, bone marrow transplant; NED, no evidence of disease.

Germline alterations included variants associated with cancer predisposition syndromes, such as *CHEK2* and Noonan syndrome *(PTPN11*). Additionally, non-cancer predisposition-associated carrier state was identified in two patients, including carrier for Cystic Fibrosis and Gaucher Disease. One variant was identified as a medically actionable finding and was associated with WHIM (Warts, Hypogammaglobulinemia, Infections, and Myelokathexis) syndrome (**Supplementary Table 6**).

Complete patient information, including somatic variants, germline findings, and results of testing prior to comprehensive genomic profiling, is available in **Supplementary Tables 3**-**5** and **Supplementary Tables 7**, **8**. **Supplementary Table 8** includes clinically significant findings. Testing done on samples prior to NGS, including cytogenetics and molecular testing, is included when available, and NGS results that were known prior to comprehensive sequencing denoted in bold.

### Patient highlights

#### IGMCH009

IGMCH0009 presented to our tertiary care center in 2015 and was diagnosed with ALCL. The patient received initial therapy per ANHL12P1 with dexamethasone, cyclophosphamide, ifosfamide, methotrexate, cytarabine, etoposide, doxorubicin, and intrathecal methotrexate/hydrocortisone and had negative end of therapy scans (98). The patient relapsed six months following diagnosis and was treated with brentuximab (anti-CD30 monoclonal antibody) for 24 cycles, achieving second remission. Approximately two years later, the patient relapsed a second time, prompting nomination and enrollment on our translational research protocol (**Figure 3B**, Image #1). RNA-sequencing identified an *NPM1::ALK* fusion. *ALK* encodes the anaplastic lymphoma kinase, a receptor tyrosine kinase belonging to the insulin receptor family (99). The *NPM1::ALK* fusion encompasses the ALK protein kinase domain and is predicted to result in overexpression, constitutive dimerization, and increased ALK kinase activity, thereby promoting tumorigenesis (100–102). The patient received brentuximab and bridging therapy with ifosfamide, carboplatin, and etoposide (ICE) followed by a matched unrelated donor hematopoietic stem cell transplant (HSCT) in 2018, achieving third remission thirty-five months following initial presentation. Third relapse was five months after the second relapse and two months after transplant (**Figure 3B**, Image #2). The patient was restarted on brentuximab and eventually transitioned to an ALK-inhibitor, crizotinib, based upon the fusion identified, achieving remission (58–60). Fourth relapse was six months after the third relapse. The patient was transitioned back to brentuximab for five months, at which point crizotinib was restarted, which is the patient’s current therapy. The patient remains without evidence of disease at last update, four years and four months since starting targeted therapy.

#### IGMCH0092

IGMCH0092 presented to our tertiary care center in 2019 at the time of second relapse of B-acute lymphoblastic leukemia (B-ALL); all prior treatment was completed at an outside hospital. Following enrollment on our translational research protocol, sequencing identified a *CD74::PDGFRB* fusion. *CD74* encodes the Cluster of Differentiation 74 protein which is involved in antigen presentation and therefore highly expressed in many hematolymphoid lineages (103). *PDGFRB* encodes a cell surface tyrosine kinase receptor for the platelet-derived growth factor family (104). This *CD74::PDGFRB* fusion is predicted to encode a chimeric protein containing the N-terminal region of CD74 fused to the transmembrane, juxtamembrane, and tyrosine kinase domains of PDGFRB, resulting in activation of downstream targets leading to leukemogenesis (105, 106). The patient received vincristine and dexamethasone bridging therapy followed by CAR-T therapy in 2019, but subsequently relapsed within one month of initiation. This third relapse was treated per AALL1131 with the addition of a tyrosine kinase inhibitor, dasatinib, based upon the fusion identified (41, 107, 108). Patient subsequently received a matched unrelated donor HSCT and remains in remission at last update, three years and 11 months since starting targeted therapy.

#### IGMCH0096

IGMCH0096 first presented to our tertiary care center in 2018 and was diagnosed with ALCL (**Figure 3C**, Image #1). The patient achieved remission with initial therapy on study per ANHL12P1, which included crizotinib (**Figure 3C**, Image #2) (98). First relapse was seven months after diagnosis, at which point the patient was nominated and enrolled on our translational research protocol **Figure 3C**, Image #3). Sequencing identified an *NPM1::ALK* fusion. Subsequent treatment included brentuximab and ICE followed by matched sibling donor HSCT two months after first relapse, achieving second remission (**Figure 3C**, Image #4). Second relapse was eight months after the first relapse, and six months after transplant (**Figure 3C**, Image #5). The patient was restarted on brentuximab and then transitioned to the ALK-inhibitor, crizotinib, which is the patient’s current therapy (58–60). The patient remains without evidence of disease at last update, three years and seven months since starting targeted therapy (**Figure 3C**, Image #6).

## Discussion

Genomic characterization of pediatric hematologic malignancies in our cohort identified potential therapeutic targets in nearly 40% of patients (7/18, 38.9%), including the consideration of inhibitors targeting MEK (n=3), receptor tyrosine kinases (ALK n=2, PDGFRB n=1), FLT3 (n=1), and PD-L1 (n=1) (28, 40, 41, 58–60, 85, 97) (**Table 1**). FLT3 inhibitors including gilteritinib and sorafenib are often used upfront in patients with *FLT3*-mutated AML. Tyrosine kinase inhibitors including dasatinib and crizotinib were implemented in three patients (3/8, 38%), all of whom remain in remission on most recent follow up, on average approximately 4 years from initiation of targeted therapy. Of note, all of these patients also underwent HSCT. However, two of these patients (IGMCH0009, IGMCH0096) relapsed after HSCT and achieved remission after starting targeted therapy. The third patient (IGMCH0092 did not have a relapse between HSCT and starting targeted therapy; remission cannot be attributed to targeted therapy alone, as HSCT likely played a large part. These results reflect the clinical impact that comprehensive molecular profiling can have on high-risk pediatric leukemia and lymphoma patients, including those that are relapsed/refractory. Although the three patients who received targeted therapy all had fusions identified, we were also able to identify patients with other sequencing variants who could potentially benefit. Further studies, more comprehensive diagnostic testing, and access to targeted treatments are needed for pediatric patients to increase use of these therapies.

Most patients within our cohort were nominated for testing because prior/standard of care treatment failed to keep them in remission and therapeutic options were desired at relapse. Of those nominated at initial diagnosis, all but one were classified as having high risk disease. Reasons for this designation included advanced stage, older or younger age, and higher risk based upon traditional testing/standard parameters.

Somatic findings were identified in most patients in this study. While some findings were previously known from cytogenetic and molecular testing, most patients with clinically significant findings had additional results from NGS that were not previously identified (**Supplementary Table 8**). In terms of diagnostic utility, somatic variants were consistent with the patient’s initial histologic diagnosis (14/18, 77.8%), which is expected, given standard testing capabilities. Importantly, however, one patient had refinement of diagnosis, which led to the use of a targeted therapy (see patient case #1). Overall, in this cohort, somatic variants were of limited prognostic utility—with somatic findings conferring clear prognostic utility in only six patients (6/18, 33.3%). This may be related to a paucity of information on genetic variants and their prognostic impact specific to pediatric malignancies, as well as the challenges to prognostication when genomic alterations confer a mixed prognosis. Furthermore, this was a diverse hematologic malignancy cohort which may limit yield in relation to our understanding of prognostic factors. These findings highlight the need for comprehensive genomics-based prognostication in pediatric hematologic malignancies (12, 109).

Germline variants led to cascade testing and additional genetic counseling in three patients in this cohort, underscoring the importance of paired germline testing for identification of cancer-predisposing variants (22, 23). Access to genetic counseling and cascade testing of at-risk individuals is critical when testing for pathogenic germline variants and can be a barrier for some institutions.

The present study does have limitations. First, the small overall number of patients combined with the varied diagnoses sequenced hampers our ability to draw broad conclusions for any singular diagnosis. Additionally, patients were selected for enrollment based on clinician nomination, rather than sequential diagnosis. While this resulted in a clinically interesting cohort that was largely comprised of high-risk patients, this provider variability may have skewed which patients were nominated and at which time point in their disease processes. Another difficulty we faced in this study was due to the implementation of paired tumor-normal exome sequencing. This methodology allows for analysis of genomic variation within the protein-coding regions of the genome, while also providing data surrounding the etiology of the variant (germline vs. somatic). Paired analysis of hematologic disease can be challenging, particularly in relation to the need for acquisition of a germline comparator sample absent of disease. Selection of germline sample comparator in patients with hematologic diseases needs to be balanced with potential drawbacks: for example, invasiveness of skin biopsies, known admixture with saliva containing disease-involved blood cells for buccal swabs, or time delay when choosing to obtain germline blood sample after initial treatment (when disease is expected to be absent or minimal) (127). Current testing strategies for risk stratification and prognostication of pediatric hematologic malignancies are not uniformly comprehensive (12, 109, 110). Widespread integration of comprehensive genomic testing with standard methodologies may lead to refined characterization of pediatric hematologic malignancies, increased understanding of known and emerging molecular drivers, and improved treatment (7, 8, 10, 11, 20, 21). Through further large-scale studies, we may elucidate additional clinically important prognostic variants, as well as new therapeutic targets, that have not previously been discovered. These findings are likely to be especially impactful in patients with relapsed or refractory disease for whom progress has lagged in improving overall survival, given current limits in treatment options and poor overall prognosis in this population (6, 17–19).

Efforts to create pediatric precision oncology programs have been undertaken nationally and internationally to evaluate the clinical benefit of NGS (1, 7–9, 20, 21, 23, 109, 111). Further studies are needed to expand on the timing of comprehensive genomic testing for pediatric hematologic malignancies and given current cost constraints, which patient cohorts should be prioritized. As more pediatric patients undergo molecular characterization of their hematologic malignancies at diagnosis and relapse, we will gain important insights affecting our understanding of genetic drivers and the overall genomic landscape of these malignancies. This knowledge can only help improve therapeutic approaches and survival in this patient population.

In conclusion, we present results from comprehensive genomic profiling of pediatric hematologic malignancies at a tertiary hospital. Our cohort of 20 patients was largely comprised of high-risk and/or relapsed patients, for whom clinicians were seeking additional molecular insights. Through comprehensive NGS of both somatic and germline samples, we identified potentially targetable alterations in 40% of patients who underwent paired sequencing (7/18). Targeted therapies were initiated in three of these patients, all of whom remain in remission an average of 47 months (nearly 4 years) post therapy initiation. While further, larger studies are needed to evaluate the applicability of these findings more broadly, our translational research highlights the importance of genomic sequencing, especially in the relapsed setting, as testing may provide effective targeted therapeutic options and prolong survival for individual patients.

## Data Availability

The datasets presented in this study can be found in online repositories. The names of the repository/repositories and accession number(s) can be found below: https://www.ncbi.nlm.nih.gov/gap/, phs001820.v3.p1.

## References

[1] Brady SW Roberts KG Gu Z Shi L Pounds S Pei D. The genomic landscape of pediatric acute lymphoblastic leukemia. Nat Genet. (2022) 54:1376–89. doi: 10.1038/s41588-022-01159-z PMC9700506 36050548

[2] Siegel RL Miller KD Fuchs HE Jemal A. Cancer statistics, 2021. CA Cancer J Clin. (2021) 71:7–33. doi: 10.3322/caac.21654 33433946

[3] Inaba H Pui CH. Advances in the diagnosis and treatment of pediatric acute lymphoblastic leukemia. J Clin Med. (2021) 10:1926. doi: 10.3390/jcm10091926 33946897 PMC8124693

[4] Frei E Holland JF Schneiderman MA Pinkel D Selkirk G Freireich EJ. A comparative study of two regimens of combination chemotherapy in acute leukemia. Blood. (1958) 13:1126–48. doi: 10.1182/blood.V13.12.1126.1126 13596417

[5] Pui CH Evans WE. A 50-year journey to cure childhood acute lymphoblastic leukemia. Semin Hematol. (2013) 50:185–96. doi: 10.1053/j.seminhematol.2013.06.007 PMC3771494 23953334

[6] Cairo MS Beishuizen A. Childhood, adolescent and young adult non-Hodgkin lymphoma: current perspectives. Br J Haematol. (2019) 185:1021–42. doi: 10.1111/bjh.15764 PMC6897376 30729513

[7] Mody RJ Wu YM Lonigro RJ Cao X Roychowdhury S Vats P. Integrative clinical sequencing in the management of refractory or relapsed cancer in youth. JAMA. (2015) 314:913–25. doi: 10.1001/jama.2015.10080 PMC4758114 26325560

[8] Parsons DW Roy A Yang Y Wang T Scollon S Bergstrom K. Diagnostic yield of clinical tumor and germline whole-exome sequencing for children with solid tumors. JAMA Oncol. (2016) 2:616–24. doi: 10.1001/jamaoncol.2015.5699 PMC5471125 26822237

[9] Marks LJ Oberg JA Pendrick D Sireci AN Glasser C Coval C. Precision medicine in children and young adults with hematologic Malignancies and blood disorders: the columbia university experience. Front Pediatr. (2017) 5:265. doi: 10.3389/fped.2017.00265 29312904 PMC5732960

[10] Mullighan CG. The molecular genetic makeup of acute lymphoblastic leukemia. Hematol Am Soc Hematol Educ Program. (2012) 2012:389–96. doi: 10.1182/asheducation-2012.1.389 23233609

[11] Vogelstein B Papadopoulos N Velculescu VE Zhou S Diaz LA Jr Kinzler KW. Cancer genome landscapes. Science. (2013) 339:1546–58. doi: 10.1126/science.1235122 PMC3749880 23539594

[12] Bolouri H Farrar JE Triche T Jr Ries RE Lim EL Alonzo TA. The molecular landscape of pediatric acute myeloid leukemia reveals recurrent structural alterations and age-specific mutational interactions. Nat Med. (2018) 24:103–12. doi: 10.1038/nm.4439 PMC5907936 29227476

[13] Den Boer ML van Slegtenhorst M De Menezes RX Cheok MH Buijs-Gladdines JG Peters ST. A subtype of childhood acute lymphoblastic leukaemia with poor treatment outcome: a genome-wide classification study. Lancet Oncol. (2009) 10:125–34. doi: 10.1016/S1470-2045(08)70339-5 PMC2707020 19138562

[14] Castellino SM Giulino-Roth L Harker-Murray P Kahn JM Forlenza C Cho S. Children's Oncology Group's 2023 blueprint for research: Hodgkin lymphoma. Pediatr Blood Cancer. (2023) 70 Suppl 6:e30580. doi: 10.1002/pbc.30580 37505794 PMC10660893

[15] Raetz EA Bhojwani D Devidas M Gore L Rabin KR Tasian SK. Children's Oncology Group blueprint for research: Acute lymphoblastic leukemia. Pediatr Blood Cancer. (2023) 70 Suppl 6:e30585. doi: 10.1002/pbc.30585 37489549 PMC10687839

[16] El-Mallawany NK Alexander S Fluchel M Hayashi RJ Lowe EJ Giulino-Roth L. Children's Oncology Group's 2023 blueprint for research: Non-Hodgkin lymphoma. Pediatr Blood Cancer. (2023) 70 Suppl 6:e30565. doi: 10.1002/pbc.30565 37449925 PMC10577684

[17] Hunger S Raetz E. How I treat relapsed acute lymphoblastic leukemia in the pediatric population. Blood. (2020) 136:1803–12. doi: 10.1182/blood.2019004043 32589723

[18] Rubnitz J Kaspers G. How I treat pediatric acute myeloid leukemia. Blood. (2021) 138:1009–18. doi: 10.1182/blood.2021011694 34115839

[19] Zarnegar-Lumley S Caldwell KJ Rubnitz JE. Relapsed acute myeloid leukemia in children and adolescents: current treatment options and future strategies. Leukemia. (2022) 36:1951–60. doi: 10.1038/s41375-022-01619-9 35668109

[20] Harris MH DuBois SG Glade Bender JL Kim A Crompton BD Parker E. Multicenter feasibility study of tumor molecular profiling to inform therapeutic decisions in advanced pediatric solid tumors: the individualized cancer therapy (iCat) study. JAMA Oncol. (2016) 2:608–15. doi: 10.1001/jamaoncol.2015.5689 26822149

[21] Summers RJ Castellino SM Porter CC MacDonald TJ Basu GD Szelinger S. Comprehensive genomic profiling of high-risk pediatric cancer patients has a measurable impact on clinical care. JCO Precis Oncol. (2022) 6:e2100451. doi: 10.1200/PO.21.00451 35544730

[22] Villani A Davidson S Kanwar N Lo WW Li Y Cohen-Gogo S. The clinical utility of integrative genomics in childhood cancer extends beyond targetable mutations. Nat Cancer. (2023) 4:203–21. doi: 10.1038/s43018-022-00474-y PMC9970873 36585449

[23] Newman S Nakitandwe J Kesserwan CA Azzato EM Wheeler DA Rusch M. Genomes for kids: the scope of pathogenic mutations in pediatric cancer revealed by comprehensive DNA and RNA sequencing. Cancer Discovery. (2021) 11:3008–27. doi: 10.1158/2159-8290.CD-20-1631 PMC8783930 34301788

[24] Roberts KG Reshmi SC Harvey RC Chen IM Patel K Stonerock E. Genomic and outcome analyses of Ph-like ALL in NCI standard-risk patients: a report from the Children's Oncology Group. Blood. (2018) 132:815–24. doi: 10.1182/blood-2018-04-841676 PMC6107876 29997224

[25] Reshmi SC Harvey RC Roberts KG Stonerock E Smith A Jenkins H. Targetable kinase gene fusions in high-risk B-ALL: a study from the Children's Oncology Group. Blood. (2017) 129:3352–61. doi: 10.1182/blood-2016-12-758979 PMC5482101 28408464

[26] Roberts KG Pei D Campana D Payne-Turner D Li Y Cheng C. Outcomes of children with BCR-ABL1–like acute lymphoblastic leukemia treated with risk-directed therapy based on the levels of minimal residual disease. J Clin Oncol. (2014) 32:3012–20. doi: 10.1200/JCO.2014.55.4105 PMC4162497 25049327

[27] Zhang J Ding L Holmfeldt L Wu G Heatley SL Payne-Turner D. The genetic basis of early T-cell precursor acute lymphoblastic leukaemia. Nature. (2012) 481:157–63. doi: 10.1038/nature10725 PMC3267575 22237106

[28] Kaburagi T Yamato G Shiba N Yoshida K Hara Y Tabuchi K. Clinical significance of RAS pathway alterations in pediatric acute myeloid leukemia. Haematologica. (2022) 107:583–92. doi: 10.3324/haematol.2020.269431 PMC8883565 33730843

[29] Jia Z Hu Z Damirchi B Han TT Gu Z. Characterization of PAX5 mutations in b progenitor acute lymphoblastic leukemia. Blood. (2022) 140:1001–2.Gen. doi: 10.1182/blood-2022-169975

[30] Liu GJ Cimmino L Jude JG Hu Y Witkowski MT McKenzie MD. Pax5 loss imposes a reversible differentiation block in B-progenitor acute lymphoblastic leukemia. Genes Dev. (2014) 28:1337–50. doi: 10.1101/gad.240416.114 PMC4066403 24939936

[31] Zhang W Kuang P Liu T. Prognostic significance of CDKN2A/B deletions in acute lymphoblastic leukaemia: a meta-analysis. Ann Med. (2019) 51:28–40. doi: 10.1080/07853890.2018.1564359 30592434 PMC7857473

[32] Sulong S Moorman AV Irving JA Strefford JC Konn ZJ Case MC. A comprehensive analysis of the CDKN2A gene in childhood acute lymphoblastic leukemia reveals genomic deletion, copy number neutral loss of heterozygosity, and association with specific cytogenetic subgroups. Blood. (2009) 113:100–7. doi: 10.1182/blood-2008-07-166801 18838613

[33] Kathiravan M Singh M Bhatia P Trehan A Varma N Sachdeva MS. Deletion of CDKN2A/B is associated with inferior relapse free survival in pediatric B cell acute lymphoblastic leukemia. Leuk Lymphoma. (2019) 60:433–41. doi: 10.1080/10428194.2018.1482542 29966470

[34] Arber DA Orazi A Hasserjian R Thiele J Borowitz MJ Le Beau MM. The 2016 revision to the World Health Organization classification of myeloid neoplasms and acute leukemia. Blood. (2016) 127:2391–405. doi: 10.1182/blood-2016-03-643544 27069254

[35] Forero-Castro M Robledo C Benito R Bodega-Mayor I Rapado I Hernández-Sánchez M. Mutations in TP53 and JAK2 are independent prognostic biomarkers in B-cell precursor acute lymphoblastic leukaemia. Br J Cancer. (2017) 117:256–65. doi: 10.1038/bjc.2017.152 PMC5520505 28557976

[36] Mullighan CG Zhang J Harvey RC Collins-Underwood JR Schulman BA Phillips LA. JAK mutations in high-risk childhood acute lymphoblastic leukemia. Proc Natl Acad Sci U S A. (2009) 106:9414–8. doi: 10.1073/pnas.0811761106 PMC2695045 19470474

[37] Mottok A Hung SS Chavez EA Woolcock B Telenius A Chong LC. Integrative genomic analysis identifies key pathogenic mechanisms in primary mediastinal large B-cell lymphoma. Blood. (2019) 134:802–13. doi: 10.1182/blood.2019001126 31292115

[38] Viganò E Gunawardana J Mottok A Van Tol T Mak K Chan FC. Somatic IL4R mutations in primary mediastinal large B-cell lymphoma lead to constitutive JAK-STAT signaling activation. Blood. (2018) 131:2036–46. doi: 10.1182/blood-2017-09-808907 29467182

[39] Steidl C Gascoyne RD. The molecular pathogenesis of primary mediastinal large B-cell lymphoma. Blood. (2011) 118:2659–69. doi: 10.1182/blood-2011-05-326538 21700770

[40] Chapuy B Stewart C Dunford AJ Kim J Wienand K Kamburov A. Genomic analyses of PMBL reveal new drivers and mechanisms of sensitivity to PD-1 blockade. Blood. (2019) 134:2369–82. doi: 10.1182/blood.2019002067 PMC6933293 31697821

[41] Weston BW Hayden MA Roberts KG Bowyer S Hsu J Fedoriw G. Tyrosine kinase inhibitor therapy induces remission in a patient with refractory EBF1-PDGFRB-positive acute lymphoblastic leukemia. J Clin Oncol. (2013) 31:e413–6. doi: 10.1200/JCO.2012.47.6770 23835704

[42] Schwab C Ryan SL Chilton L Elliott A Murray J Richardson S. EBF1-PDGFRB fusion in pediatric B-cell precursor acute lymphoblastic leukemia (BCP-ALL): genetic profile and clinical implications. Blood. (2016) 127:2214–8. doi: 10.1182/blood-2015-09-670166 26872634

[43] Wang Q Qiu H Jiang H Wu L Dong S Pan J. Mutations of PHF6 are associated with mutations of NOTCH1, JAK1 and rearrangement of SET-NUP214 in T-cell acute lymphoblastic leukemia. Haematologica. (2011) 96:1808–14. doi: 10.3324/haematol.2011.043083 PMC3232263 21880637

[44] Van Vlierberghe P Palomero T Khiabanian H van der Meulen J Castillo M Van Roy N. PHF6 mutations in T-cell acute lymphoblastic leukemia. Nat Genet. (2010) 42:338–42. doi: 10.1038/ng.542 PMC2847364 20228800

[45] Kurzer JH Weinberg OK. *PHF6* mutations in hematologic Malignancies. Front Oncol. (2021) 11:704471. doi: 10.3389/fonc.2021.704471 34381727 PMC8350393

[46] Krizsán S Péterffy B Egyed B Nagy T Sebestyén E Hegyi LL. Next-generation sequencing-based genomic profiling of children with acute myeloid leukemia. J Mol Diagn. (2023) 25:555–68. doi: 10.1016/j.jmoldx.2023.04.004 PMC10435843 37088137

[47] Li M Collins R Jiao Y Ouillette P Bixby D Erba H. Somatic mutations in the transcriptional corepressor gene BCORL1 in adult acute myelogenous leukemia. Blood. (2011) 118:5914–7. doi: 10.1182/blood-2011-05-356204 PMC3228503 21989985

[48] de Rooij JD van den Heuvel-Eibrink MM Hermkens MC Verboon LJ Arentsen-Peters ST Fornerod M. BCOR and BCORL1 mutations in pediatric acute myeloid leukemia. Haematologica. (2015) 100:e194–5. doi: 10.3324/haematol.2014.117796 PMC4420230 25596268

[49] Balgobind BV Van Vlierberghe P van den Ouweland AM Beverloo HB Terlouw-Kromosoeto JN van Wering ER. Leukemia-associated NF1 inactivation in patients with pediatric T-ALL and AML lacking evidence for neurofibromatosis. Blood. (2008) 111:4322–8. doi: 10.1182/blood-2007-06-095075 18172006

[50] Ney GM Anderson B Bender J Kumar-Sinha C Wu YM Vats P. Mutations predictive of hyperactive Ras signaling correlate with inferior survival across high-risk pediatric acute leukemia. Transl Pediatr. (2020) 9:43–50. doi: 10.21037/tp.2019.12.03 32154134 PMC7036640

[51] Rampal R Figueroa ME. Wilms tumor 1 mutations in the pathogenesis of acute myeloid leukemia. Haematologica. (2016) 101:672–9. doi: 10.3324/haematol.2015.141796 PMC5013955 27252512

[52] Pratcorona M Abbas S Sanders MA Koenders JE Kavelaars FG Erpelinck-Verschueren CA. Acquired mutations in ASXL1 in acute myeloid leukemia: prevalence and prognostic value. Haematologica. (2012) 97:388–92. doi: 10.3324/haematol.2011.051532 PMC3291593 22058207

[53] Paschka P Schlenk RF Gaidzik VI Herzig JK Aulitzky T Bullinger L. ASXL1 mutations in younger adult patients with acute myeloid leukemia: a study by the German-Austrian Acute Myeloid Leukemia Study Group. Haematologica. (2015) 100:324–30. doi: 10.3324/haematol.2014.114157 PMC4349270 25596267

[54] Ding LW Sun QY Tan KT Chien W Mayakonda A Yeoh AEJ. Mutational landscape of pediatric acute lymphoblastic leukemia. Cancer Res. (2017) 77:390–400. doi: 10.1158/0008-5472.CAN-16-1303 27872090 PMC5243866

[55] Savage NM Kota V Manaloor EJ Kulharya AS Pierini V Mecucci C. Acute leukemia with PICALM-MLLT10 fusion gene: diagnostic and treatment struggle. Cancer Genet Cytogenet. (2010) 202:129–32. doi: 10.1016/j.cancergencyto.2010.07.126 20875875

[56] Caudell D Aplan PD. The role of CALM-AF10 gene fusion in acute leukemia. Leukemia. (2008) 22:678–85. doi: 10.1038/sj.leu.2405074 PMC2366104 18094714

[57] Borel C Dastugue N Cances-Lauwers V Mozziconacci MJ Prebet T Vey N. PICALM-MLLT10 acute myeloid leukemia: a French cohort of 18 patients. Leuk Res. (2012) 36:1365–9. doi: 10.1016/j.leukres.2012.07.008 22871473

[58] Krumbholz M Woessmann W Zierk J Seniuk D Ceppi P Zimmermann M. Characterization and diagnostic application of genomic *NPM-ALK* fusion sequences in anaplastic large-cell lymphoma. Oncotarget. (2018) 9:26543–55. doi: 10.18632/oncotarget.25489 PMC5995187 29899875

[59] Werner MT Zhao C Zhang Q Wasik MA. Nucleophosmin-anaplastic lymphoma kinase: the ultimate oncogene and therapeutic target. Blood. (2017) 129:823–31. doi: 10.1182/blood-2016-05-717793 27879258

[60] Werner MT Zhang Q Wasik MA. From pathology to precision medicine in anaplastic large cell lymphoma expressing anaplastic lymphoma kinase (ALK+ ALCL). Cancers (Basel). (2017) 9:138. doi: 10.3390/cancers9100138 29035291 PMC5664077

[61] Schmitz R Young RM Ceribelli M Jhavar S Xiao W Zhang M. Burkitt lymphoma pathogenesis and therapeutic targets from structural and functional genomics. Nature. (2012) 490:116–20. doi: 10.1038/nature11378 PMC3609867 22885699

[62] Thomas N Dreval K Gerhard DS Hilton LK Abramson JS Ambinder RF. Genetic subgroups inform on pathobiology in adult and pediatric Burkitt lymphoma. Blood. (2023) 141:904–16. doi: 10.1182/blood.2022016534 PMC10023728 36201743

[63] Panea RI Love CL Shingleton JR Reddy A Bailey JA Moormann AM. The whole-genome landscape of Burkitt lymphoma subtypes. Blood. (2019) 134:1598–607. doi: 10.1182/blood.2019001880 PMC6871305 31558468

[64] Li B Brady SW Ma X Shen S Zhang Y Li Y. Therapy-induced mutations drive the genomic landscape of relapsed acute lymphoblastic leukemia. Blood. (2020) 135:41–55. doi: 10.1182/blood.2019002220 31697823 PMC6940198

[65] Ma X Edmonson M Yergeau D Muzny DM Hampton OA Rusch M. Rise and fall of subclones from diagnosis to relapse in pediatric B-acute lymphoblastic leukaemia. Nat Commun. (2015) 6:6604. doi: 10.1038/ncomms7604 25790293 PMC4377644

[66] Sakai I Tamura T Narumi H Uchida N Yakushijin Y Hato T. Novel RUNX1-PRDM16 fusion transcripts in a patient with acute myeloid leukemia showing t(1;21)(p36;q22). Genes Chromosomes Cancer. (2005) 44:265–70. doi: 10.1002/gcc.20241 16015645

[67] Stevens-Kroef MJ Schoenmakers E van Kraaij M Huys E Vermeulen S van der Reijden B. Identification of truncated *RUNX1* and *RUNX1-PRDM16* fusion transcripts in a case of t(1;21)(p36;q22)-positive therapy-related AML. Leukemia. (2006) 20:1187–9. doi: 10.1038/sj.leu.2404210 16598304

[68] Steidl C Shah SP Woolcock BW Rui L Kawahara M Farinha P. MHC class II transactivator CIITA is a recurrent gene fusion partner in lymphoid cancers. Nature. (2011) 471:377–81. doi: 10.1038/nature09754 PMC3902849 21368758

[69] Mottok A Woolcock B Chan FC Tong KM Chong L Farinha P. Genomic alterations in CIITA are frequent in primary mediastinal large B cell lymphoma and are associated with diminished MHC class II expression. Cell Rep. (2015) 13:1418–31. doi: 10.1016/j.celrep.2015.10.008 26549456

[70] Wagner SD Ahearne M Ko Ferrigno P. The role of BCL6 in lymphomas and routes to therapy. Br J Haematol. (2011) 152:3–12. doi: 10.1111/j.1365-2141.2010.08420.x 21083654

[71] Love C Sun Z Jima D Li G Zhang J Miles R. The genetic landscape of mutations in Burkitt lymphoma. Nat Genet. (2012) 44:1321–5. doi: 10.1038/ng.2468 PMC3674561 23143597

[72] López C Kleinheinz K Aukema SM Rohde M Bernhart SH Hübschmann D. Genomic and transcriptomic changes complement each other in the pathogenesis of sporadic Burkitt lymphoma. Nat Commun. (2019) 10:1459. doi: 10.1038/s41467-019-08578-3 30926794 PMC6440956

[73] Sun R Medeiros LJ Young KH. Diagnostic and predictive biomarkers for lymphoma diagnosis and treatment in the era of precision medicine. Mod Pathol. (2016) 29:1118–42. doi: 10.1038/modpathol.2016.92 27363492

[74] Rohde M Bonn BR Zimmermann M Lange J Möricke A Klapper W. Relevance of ID3-TCF3-CCND3 pathway mutations in pediatric aggressive B-cell lymphoma treated according to the non-Hodgkin Lymphoma Berlin-Frankfurt-Münster protocols. Haematologica. (2017) 102:1091–8. doi: 10.3324/haematol.2016.156885 PMC5451341 28209658

[75] Breit S Stanulla M Flohr T Schrappe M Ludwig WD Tolle G. Activating NOTCH1 mutations predict favorable early treatment response and long-term outcome in childhood precursor T-cell lymphoblastic leukemia. Blood. (2006) 108:1151–7. doi: 10.1182/blood-2005-12-4956 16614245

[76] Kubota-Tanaka M Osumi T Miura S Tsujimoto H Imamura T Nishimura A. B-lymphoblastic lymphoma with *TCF3-PBX1* fusion gene. Haematologica. (2019) 104:e35–7. doi: 10.3324/haematol.2018.199885 PMC6312003 30262566

[77] Haas OA Borkhardt A. Hyperdiploidy: the longest known, most prevalent, and most enigmatic form of acute lymphoblastic leukemia in children. Leukemia. (2022) 36:2769–83. doi: 10.1038/s41375-022-01720-z PMC9712104 36266323

[78] Bentz M Barth TF Brüderlein S Bock D Schwerer MJ Baudis M. Gain of chromosome arm 9p is characteristic of primary mediastinal B-cell lymphoma (MBL): comprehensive molecular cytogenetic analysis and presentation of a novel MBL cell line. Genes Chromosomes Cancer. (2001) 30:393–401. doi: 10.1002/1098-2264(2001)9999:9999<::aid-gcc1105>3.0.co;2-i 11241792

[79] Chen H Pan T He Y Zeng R Li Y Yi L. Primary mediastinal B-cell lymphoma: novel precision therapies and future directions. Front Oncol. (2021) 11:654854. doi: 10.3389/fonc.2021.654854 33869061 PMC8044947

[80] Schmitz R Hansmann ML Bohle V Martin-Subero JI Hartmann S Mechtersheimer G. TNFAIP3 (A20) is a tumor suppressor gene in Hodgkin lymphoma and primary mediastinal B cell lymphoma. J Exp Med. (2009) 206:981–9. doi: 10.1084/jem.20090528 PMC2715030 19380639

[81] Jia Z Gu Z. PAX5 alterations in B-cell acute lymphoblastic leukemia. Front Oncol. (2022) 12:1023606. doi: 10.3389/fonc.2022.1023606 36387144 PMC9640836

[82] Górecki M Kozioł I Kopystecka A Budzyńska J Zawitkowska J Lejman M. Updates in KMT2A gene rearrangement in pediatric acute lymphoblastic leukemia. Biomedicines. (2023) 11:821. doi: 10.3390/biomedicines11030821 36979800 PMC10045821

[83] Choi S Kim BK Ahn HY Hong KT Choi JY Shin HY. Outcomes of pediatric acute myeloid leukemia patients with FLT3-ITD mutations in the pre-FLT3 inhibitor era. Blood Res. (2020) 55:217–24. doi: 10.5045/br.2020.2020127 PMC7784129 33232940

[84] Qiu KY Liao XY Liu Y Huang K Li Y Fang JP. Poor outcome of pediatric patients with acute myeloid leukemia harboring high FLT3/ITD allelic ratios. Nat Commun. (2022) 13:3679. doi: 10.1038/s41467-022-31489-9 35760968 PMC9237020

[85] McCall D Roth M Mahadeo KM Toepfer L Nunez C Short NJ. Gilteritinib combination therapies in pediatric patients with FLT3-mutated acute myeloid leukemia. Blood Adv. (2021) 5:5215–9. doi: 10.1182/bloodadvances.2021005164 PMC9153013 34592761

[86] Gelsi-Boyer V Brecqueville M Devillier R Murati A Mozziconacci MJ Birnbaum D. Mutations in ASXL1 are associated with poor prognosis across the spectrum of Malignant myeloid diseases. J Hematol Oncol. (2012) 5:12. doi: 10.1186/1756-8722-5-12 22436456 PMC3355025

[87] Schnittger S Eder C Jeromin S Alpermann T Fasan A Grossmann V. ASXL1 exon 12 mutations are frequent in AML with intermediate risk karyotype and are independently associated with an adverse outcome. Leukemia. (2013) 27:82–91. doi: 10.1038/leu.2012.262 23018865

[88] Gu Z Churchman ML Roberts KG Moore I Zhou X Nakitandwe J. PAX5-driven subtypes of B-progenitor acute lymphoblastic leukemia. Nat Genet. (2019) 51:296–307. doi: 10.1038/s41588-018-0315-5 30643249 PMC6525306

[89] Jung M Schieck M Hofmann W Tauscher M Lentes J Bergmann A. Frequency and prognostic impact of PAX5 p.P80R in pediatric acute lymphoblastic leukemia patients treated on an AIEOP-BFM acute lymphoblastic leukemia protocol. Genes Chromosomes Cancer. (2020) 59:667–71. doi: 10.1002/gcc.22882 PMC7540392 32592278

[90] Barragán E Cervera J Bolufer P Ballester S Martín G Fernández P. Prognostic implications of Wilms' tumor gene (WT1) expression in patients with *de novo* acute myeloid leukemia. Haematologica. (2004) 89:926–33. 15339675

[91] Bergmann L Miething C Maurer U Brieger J Karakas T Weidmann E. High levels of Wilms' tumor gene (wt1) mRNA in acute myeloid leukemias are associated with a worse long-term outcome. Blood. (1997) 90:1217–25. doi: 10.1182/blood.V90.3.1217 9242555

[92] Pan X Mengge G Wang K Wang Y Kong J Sun Y. Prognostic impact of WT1 mutation on AML of different risk groups based on 2022 European leukemianet (ELN) risk classification. Blood. (2022) 140:3216–7. doi: 10.1182/blood-2022-166323

[93] Hof J Szymansky A Stackelberg Av Eckert C Kirschner-Schwabe R. Clinical significance of NT5C2 mutations in children with first relapse of B-cell precursor acute lymphoblastic leukemia. Blood. (2014) 124:3789. doi: 10.1182/blood.V124.21.3789.3789

[94] Dieck CL Tzoneva G Forouhar F Carpenter Z Ambesi-Impiombato A Sánchez-Martín M. Structure and mechanisms of NT5C2 mutations driving thiopurine resistance in relapsed lymphoblastic leukemia. Cancer Cell. (2018) 34:136–147.e6. doi: 10.1016/j.ccell.2018.06.003 29990496 PMC6049837

[95] Dieck CL Ferrando A. Genetics and mechanisms of NT5C2-driven chemotherapy resistance in relapsed ALL. Blood. (2019) 133:2263–8. doi: 10.1182/blood-2019-01-852392 PMC6533602 30910786

[96] Barz MJ Hof J Groeneveld-Krentz S Loh JW Szymansky A Astrahantseff K. Subclonal NT5C2 mutations are associated with poor outcomes after relapse of pediatric acute lymphoblastic leukemia. Blood. (2020) 135:921–33. doi: 10.1182/blood.2019002499 PMC7218751 31971569

[97] Jerchel IS Hoogkamer AQ Ariës IM Steeghs EMP Boer JM Besselink NJM. RAS pathway mutations as a predictive biomarker for treatment adaptation in pediatric B-cell precursor acute lymphoblastic leukemia. Leukemia. (2018) 32:931–40. doi: 10.1038/leu.2017.303 PMC5886052 28972594

[98] Lowe EJ Reilly AF Lim MS Gross TG Saguilig L Barkauskas DA. Crizotinib in combination with chemotherapy for pediatric patients with ALK+ Anaplastic large-cell lymphoma: the results of children's oncology group trial ANHL12P1. J Clin Oncol. (2023) 41:2043–53. doi: 10.1200/JCO.22.00272 PMC10082271 36534942

[99] Della Corte CM Viscardi G Di Liello R Fasano M Martinelli E Troiani T. Role and targeting of anaplastic lymphoma kinase in cancer. Mol Cancer. (2018) 17:30. doi: 10.1186/s12943-018-0776-2 29455642 PMC5817803

[100] Rodrigues GA Park M. Oncogenic activation of tyrosine kinases. Curr Opin Genet Dev. (1994) 4:15–24. doi: 10.1016/0959-437x(94)90086-8 8193535

[101] Bischof D Pulford K Mason DY Morris SW. Role of the nucleophosmin (NPM) portion of the non-Hodgkin's lymphoma-associated NPM-anaplastic lymphoma kinase fusion protein in oncogenesis. Mol Cell Biol. (1997) 17:2312–25. doi: 10.1128/MCB.17.4.2312 PMC232080 9121481

[102] Hernández L Beà S Bellosillo B Pinyol M Falini B Carbone A. Diversity of genomic breakpoints in TFG-ALK translocations in anaplastic large cell lymphomas: identification of a new TFG-ALK(XL) chimeric gene with transforming activity. Am J Pathol. (2002) 160:1487–94. doi: 10.1016/S0002-9440(10)62574-6 PMC1867210 11943732

[103] Vargas J Pantouris G. Analysis of CD74 occurrence in oncogenic fusion proteins. Int J Mol Sci. (2023) 24:15981. doi: 10.3390/ijms242115981 37958963 PMC10650716

[104] Demoulin JB Montano-Almendras CP. Platelet-derived growth factors and their receptors in normal and Malignant hematopoiesis. Am J Blood Res. (2012) 2:44–56. 22432087 PMC3301440

[105] Welsh SJ Churchman ML Togni M Mullighan CG Hagman J. Deregulation of kinase signaling and lymphoid development in EBF1-PDGFRB ALL leukemogenesis. Leukemia. (2018) 32:38–48. doi: 10.1038/leu.2017.166 28555080 PMC5709252

[106] Sadras T Jalud FB Kosasih HJ Horne CR Brown LM El-Kamand S. Unusual PDGFRB fusion reveals novel mechanism of kinase activation in Ph-like B-ALL. Leukemia. (2023) 37:905–9. doi: 10.1038/s41375-023-01843-x PMC10079538 36810896

[107] Lengline E Beldjord K Dombret H Soulier J Boissel N Clappier E. Successful tyrosine kinase inhibitor therapy in a refractory B-cell precursor acute lymphoblastic leukemia with EBF1-PDGFRB fusion. Haematologica. (2013) 98:e146–8. doi: 10.3324/haematol.2013.095372 PMC3815191 24186319

[108] Salzer WL Burke MJ Devidas M Dai Y Hardy KK Kairalla JA. Impact of intrathecal triple therapy versus intrathecal methotrexate on disease-free survival for high-risk B-lymphoblastic leukemia: children's oncology group study AALL1131. J Clin Oncol. (2020) 38:2628–38. doi: 10.1200/JCO.19.02892 PMC7402996 32496902

[109] Umeda M Ma J Westover T Ni Y Song G Maciaszek JL. A new genomic framework to categorize pediatric acute myeloid leukemia. Nat Genet. (2024) 56:281–93. doi: 10.1038/s41588-023-01640-3 PMC10864188 38212634

[110] Farrar JE Schuback HL Ries RE Wai D Hampton OA Trevino LR. Genomic profiling of pediatric acute myeloid leukemia reveals a changing mutational landscape from disease diagnosis to relapse. Cancer Res. (2016) 76:2197–205. doi: 10.1158/0008-5472.CAN-15-1015 PMC4873364 26941285

[111] Langenberg KPS Looze EJ Molenaar JJ. The landscape of pediatric precision oncology: program design, actionable alterations, and clinical trial development. Cancers (Basel). (2021) 13:4324. doi: 10.3390/cancers13174324 34503139 PMC8431194

[112] Kelly BJ Fitch JR Hu Y Corsmeier DJ Zhong H Wetzel AN. Churchill: an ultra-fast, deterministic, highly scalable and balanced parallelization strategy for the discovery of human genetic variation in clinical and population-scale genomics. Genome Biol. (2015) 16:6. doi: 10.1186/s13059-014-0577-x 25600152 PMC4333267

[113] Van der Auwera GOCB. Genomics in the Cloud: Using Docker, GATK, and WDL in 1st Edition. Sebastopol: O'Reilly Media, Inc (2020).

[114] Poplin R Ruano-Rubio V DePristo M Fennell T Carneiro M van der Auwera G. Scaling accurate genetic variant discovery to tens of thousands of samples. BioRxiv. (2018). doi: 10.1101/201178

[115] Cibulskis K Lawrence MS Carter SL Sivachenko A Jaffe D Sougnez C. Sensitive detection of somatic point mutations in impure and heterogeneous cancer samples. Nat Biotechnol. (2013) 31:213–9. doi: 10.1038/nbt.2514 PMC3833702 23396013

[116] Koboldt DC Zhang Q Larson DE Shen D McLellan MD Lin L. VarScan 2: somatic mutation and copy number alteration discovery in cancer by exome sequencing. Genome Res. (2012) 22:568–76. doi: 10.1101/gr.129684.111 PMC3290792 22300766

[117] Zhang J Walsh MF Wu G Edmonson MN Gruber TA Easton J. Germline mutations in predisposition genes in pediatric cancer. N Engl J Med. (2015) 373:2336–46. doi: 10.1056/NEJMoa1508054 PMC4734119 26580448

[118] Sondka Z Bamford S Cole CG Ward SA Dunham I Forbes SA. The COSMIC Cancer Gene Census: describing genetic dysfunction across all human cancers. Nat Rev Cancer. (2018) 18:696–705. doi: 10.1038/s41568-018-0060-1 30293088 PMC6450507

[119] LaHaye S Fitch JR Voytovich KJ Herman AC Kelly BJ Lammi GE. Discovery of clinically relevant fusions in pediatric cancer. BMC Genomics. (2021) 22:872. doi: 10.1186/s12864-021-08094-z 34863095 PMC8642973

[120] Haas BJ Dobin A Li B Stransky N Pochet N Regev A. Accuracy assessment of fusion transcript detection via read-mapping and *de novo* fusion transcript assembly-based methods. Genome Biol. (2019) 20:213. doi: 10.1186/s13059-019-1842-9 31639029 PMC6802306

[121] Wang K Singh D Zeng Z Coleman SJ Huang Y Savich GL. MapSplice: accurate mapping of RNA-seq reads for splice junction discovery. Nucleic Acids Res. (2010) 38:e178. doi: 10.1093/nar/gkq622 20802226 PMC2952873

[122] Nicorici D Satalan M Edgren H Kangaspeska S Murumagi A Kallioniemi O. FusionCatcher - a tool for finding somatic fusion genes in paired-end RNA-sequencing data. bioRxiv. (2014). doi: 10.1101/011650

[123] Ge H Liu K Juan T Fang F Newman M Hoeck W. FusionMap: detecting fusion genes from next-generation sequencing data at base-pair resolution. Bioinformatics. (2011) 27:1922–8. doi: 10.1093/bioinformatics/btr310 21593131

[124] Davidson NM Majewski IJ Oshlack A. JAFFA: High sensitivity transcriptome-focused fusion gene detection. Genome Med. (2015) 7:43. doi: 10.1186/s13073-015-0167-x 26019724 PMC4445815

[125] Tian L Li Y Edmonson MN Zhou X Newman S McLeod C. CICERO: a versatile method for detecting complex and diverse driver fusions using cancer RNA sequencing data. Genome Biol. (2020) 21:126. doi: 10.1186/s13059-020-02043-x 32466770 PMC7325161

[126] Uhrig S Ellermann J Walther T Burkhardt P Frohlich M Hutter B. Accurate and efficient detection of gene fusions from RNA sequencing data. Genome Res. (2021) 31:448–60p. doi: 10.1101/gr.257246.119 33441414 PMC7919457

[127] Ptashkin RN Ewalt MD Jayakumaran G Kiecka I Bowman AS Yao J. Enhanced clinical assessment of hematologic Malignancies through routine paired tumor and normal sequencing. Nat Commun. (2023) 14:6895. doi: 10.1038/s41467-023-42585-9 37898613 PMC10613284

