# Supplementary material for: Comprehensive genomic characterization of hematologic malignancies at a pediatric tertiary care center
Source: Front Oncol. 2024 Dec 2;14:1498409. doi: 10.3389/fonc.2024.1498409 (PMC11647012; doi:10.3389/fonc.2024.1498409)
Supplement: Supplementary file 1 [file SupplementaryFile1.docx]

**Supplementary Material Sources**

1. Gupta S, Rau R, Rabin K. A Phase 3 Trial Investigating Blinatumomab (IND# 117467, NSC# 765986) in Combination with Chemotherapy in Patients with Newly Diagnosed Standard Risk or Down syndrome B-Lymphoblastic Leukemia (B-ALL) and the Treatment of Patients with Localized B-Lymphoblastic Lymphoma (B-LLy): A COG Groupwide Phase III Study. 2019 June 28- 2024 August 16. Accessed 2024 October 21.
2. Cooper TM, Pollard J. A Phase 3 Randomized Trial for Patients with de novo AML Comparing Standard Therapy Including Gemtuzumab Ozogamicin (GO) to CPX-351 with GO, and the Addition of the FLT3 Inhibitor Gilteritinib for Patients with FLT3 Mutations: A COG Groupwide Phase 3 Study. 2020 July 20- current. Accessed 2024 October 21.
