# Supplementary material for: Comprehensive genomic characterization of hematologic malignancies at a pediatric tertiary care center
Source: Front Oncol. 2024 Dec 2;14:1498409. doi: 10.3389/fonc.2024.1498409 (PMC11647012; doi:10.3389/fonc.2024.1498409)
Supplement: Supplementary file 2 [file Table1.docx]

**Supplementary Tables:**

**Supplementary Table 1. Demographics**

| **Characteristic** | **Patients (%)** |
| --- | --- |
| **Diagnosis** |  |
| Non-Hodgkin Lymphoma (NHL) | 5 (27.8%) |
| Anaplastic Large Cell Lymphoma (ALCL) | 2 (11.1%) |
| Burkitt Lymphoma/Leukemia | 2 (11.1%) |
| Primary Mediastinal B Cell Lymphoma (PMBCL) | 1 (5.5%) |
| Hodgkin Lymphoma (HL) | 1 (5.5%) |
| Acute Myeloid Leukemia (AML) | 3 (16.7%) |
| Acute Lymphoblastic Leukemia (ALL) | 9 (50%) |
| Infant | 1 (5.5%) |
| Non-infant | 8 (44.5%) |
| **Age at Nomination** |  |
| 0-5 years | 5 (27.8%) |
| 6-10 years | 4 (22.2%) |
| 11-15 years | 5 (27.8%) |
| 16-20 years | 2 (11.1%) |
| 21-25 years | 1 (5.5%) |
| 26-30 years | 1 (5.5%) |
| **Time Followed** |  |
| 0-5 years | 13 (72.2%) |
| 6-10 years | 3 (16.7%) |
| 11-15 years | 1 (5.5%) |
| >15 years | 1 (5.5%) |
| **Race** |  |
| White | 13 (72.2%) |
| Black | 2 (11.1%) |
| Asian | 1 (5.5%) |
| Multi-racial | 2 (11.1%) |
| **Sex** |  |
| Female | 5 (27.8%) |
| Male | 13 (72.2%) |
| **Status at Nomination** |  |
| New Diagnosis | 7 (38.9%) |
| Relapse | 9 (50%) |
| Remission | 2 (11.1) |
| **Status at last update** |  |
| Alive | 14 (77.8%) |
| No Evidence of Disease | 13 (72.2%) |
| Progressive Disease | 1 (5.5%) |
| Deceased | 4 (22.2%) |

**Supplementary Table 2. Reason for Testing**

| **Reason** | **Number of Patients (N=18)** |
| --- | --- |
| Relapsed/Refractory Disease | 9 (50%) |
| Multiple Malignancies | 2 (11.1%) |
| Poor Prognosis | 2 (11.1%) |
| Confirm Diagnosis | 2 (11.1%) |
| Neurologic Symptoms | 1 (5.6%) |
| Therapeutic Toxicity | 1 (5.6%) |
| Presentation | 1 (5.6%) |

**Supplementary Table 3. Diagnostic Findings**

| **Patient**  **(IGMCH#)** | **Diagnosis** | **Somatic Variant** | **Type of Variant** | **Impact on Diagnosis** |
| --- | --- | --- | --- | --- |
| **0009** | ALCL | *NPM**1::ALK* (NM_002520, NM_004304)^52-53, 86-87^ | Fusion | Refined |
| **0056** | T-ALL | *PHF6* c.309C>A(p.Tyr103Ter) (NM_032458.2)^43-45^  *JAK1* c.2108G>T(p.Ser703Ile) (NM_001321854.1)^36, 54^  *STAT5B* c.1924A>C(p.Asn642His) (NM_012448.3)^38^ | SNV  SNV  SNV | Confirmed |
| **0086** | B-ALL | *PAX5* c.80T>C(p.Phe27Ser) (NM_001280555.1)^29-30, 81^  *PA*X5 LOH^29-30, 81^  *CDKN2A* loss^31-33^  *ZCCHC**7*::*PAX5* (NM_001289119, NM_001280555)^81^ | SNV  CNV  CNV  Fusion | Confirmed |
| **0089** | B-ALL | *PAX5* c.239C>G(p.Pro80Arg) (NM_016734)^29-30, 81^  *TP53* c.526T>C(p.Cys176Arg) (NM_000546)^35^  *PAX5* loss^29-30, 81^  *CDKN2A* loss^31-33^  *TP53* loss^7^  *TP53* c.846_847insAGCAGGG(p.Arg283SerfsTer25) (NM_000546)^35^ | SNV  SNV  CNV  CNV  CNV  Indel | Confirmed |
| **0092** | B-ALL | *CDKN2A* loss^31-33^  *CD**74::PDGFRB*^41-42^ | CNV  Fusion | Confirmed |
| **0093** | AML | *PHF6* c.986A>G(p.His329Arg) (NM_001015877.1)^43-45^  *BCORL1* c.4258C>T(p.Arg1420Ter) (NM_021946.4)^46-48^  *WT1* c.1319G>Ap.Arg440Lys (NM_024426.4)^51^  *PICALM::MLLT10* (NM_007166.4, NM_004641.3)^55-57^  *NF1* c.402_406delTCGGAinsCTTT(p.Arg135PhefsTer30) (NM_000267.3:)^28, 49-50^  *NF1* c.6925_6926insTCTGGGGGGGC(p.Ser2309PhefsTer14) (NM_001042492.2)^28, 49-50^  *ASXL1* c.1933_1934dupGG(p.Gly646ValfsTer58) (NM_015338.5)^52-53^  *CREBBP* deletion exons 7-13 Chr 16 (NM_004380.2)^54^ | SNV  SNV  SNV  Fusion  Indel  Indel  Indel  Indel | Confirmed |
| **0096** | ALCL | *NPM**1*::*ALK* (NM_002520.6, NM_004304.5)^58-60^ | Fusion | Confirmed |
| **0120** | B-ALL | *CDKN2A* loss^31-33^  *KMT2**A*::*MLLT4*^54, 82^ | CNV  Fusion | Confirmed |
| **0194** | BL and NBL | *DDX3X* c.79C>T(p.Gln27Ter) (NM_001356.4)^61-63^  *IGLL5*::*PVT1* (NM_001256296.2, NR_003367.3)^61-63^ | SNV  Fusion | Confirmed |
| **0242** | B-ALL | *NSD2* c.3448A>G(p.Thr1150Ala) (NM_001042424.3)^64-65^  *CDKN2A* loss^31-33^  Chromosome 6p gain^54, 77^  *TCF**3*::*PBX1* (NM_003200.5, NM_002585.4)^34^ | SNV  CNV  CNV  Fusion | Confirmed |
| **0252** | AML and WT | *KRAS* c.37G>T(p.Gly13Cys) (NM_033360.4>NM_033360.3)^24-27^  *RUNX**1*::*PRDM16* (NM_001754.4, NM_022114.4)^66-67^  *ASXL1* c.1900_1922delAGAGAGGCGGCCACCACTGCCAT(p.Glu635ArgfsTer15) (NM_015338.6>NM_015338.5)^52-53^  *FLT3* ITD (M_004119.3>NM_004119.2)^83-85^ | SNV  Fusion  Indel  Internal Tandem Duplication | Confirmed |
| **0300** | PMBCL | *SOCS1* c.574G>T(p.Ala192Ser) (NM_003745.2)^37, 39-40^  *SOCS1* c.615C>G(p.Ser205Arg) (NM_003745.2)^37, 39-40^  *JAK1* c.1872A>T(p.Leu624Phe) (NM_002227.4)^37-40^  *IL4R* c.127A>C(p.Thr43Pro) (NM_000418.4)^37-38, 40^  *IL4R* c.598C>T(p.Arg200Trp) (NM_000418.4)^37-38, 40^  *IRF2BP2* c.295C>T(p.Leu99Phe) (NM_182972.3)^37, 40^  *SOCS1* loss^37, 39-40^  *B2M* loss^40^  Chromosome 6 loss^79-80^  Chromosome 9 gain^37-40, 78^  *CIITA*::*RMI2* (NM_000246.3, NM_152308.3)^37, 68-69^  *B2M* c.165delT(p.Ile55MetfsTer6) (NM_004048.4)^40^ | SNV  SNV  SNV  SNV  SNV  SNV  CNV  CNV  CNV  CNV  Fusion  Indel | Confirmed |
| **0329** | Infant B-ALL | *KRAS* c.35G>A(p.Gly12Asp) (NM_004985.5)^37, 39-40^ | SNV | Confirmed |
| **0371** | B-ALL | Chromosome 7p loss^54, 77^  Chromosomes 5, 6, 7q, 10, 14, 17, 18, 21, 22, X gains^54, 77^ | CNV  CNV | Confirmed |
| **0423** | Burkitt Leukemia | *DDX3X* c.1583G>A(p.Arg528His) (NM_001356.5)^61-63^  *BCL6* c.1760C>A(p.Ala587Asp) (NM_001706.5)^63, 70^  *SMARCA4* c.3574Cc>T(p.Arg1192Cys) (NM_003072.5)^61, 71-72^  *ID3* c.166C>T(p.Pro56Ser) (NM_002167.5)^61, 63, 71-74^ | SNV  SNV  SNV  SNV | Confirmed |

ALCL – anaplastic large cell lymphoma; T-ALL – T-cell acute lymphoblastic leukemia; B-ALL – B-cell acute lymphoblastic leukemia; AML – acute myeloid leukemia; BL – Burkitt Lymphoma; NBL – Neuroblastoma; WT – Wilm's Tumor; PMBCL – primary mediastinal B cell lymphoma; SNV – single nucleotide variant; indel – insertion/deletion; CNV – copy number variant; ITD – internal tandem duplication

**Supplementary Table 4. Testing Results prior to Comprehensive Genomic Profiling**

| **Patient (IGMCH#)** | **Karyotyping Results** | **FISH Results** | **Molecular Results** |
| --- | --- | --- | --- |
| **0009** | Bone Marrow (left): 46,XX [20]  Bone Marrow (right): 46,XX[20]  Lymph Node: N/A- karyotyping not completed on sample | Bone Marrow (left and right): Negative for ALK rearrangement; nuc ish(ALKx2)[200]  Lymph Node: N/A- FISH not completed on sample | none |
| **0056** | Peripheral Blood: N/A-no dividing cells | Peripheral Blood: nuc ish(CDKN2Ax1, D9Z4x2)[195/205], (KMT2Ax1)[191/200], (TRBx2)(200), (BCR, ABL1)x2[200], (TCL1x2)[200] | none |
| **0086** | Bone Marrow: 46, XY[20] | Bone Marrow: nuc ish (D4Z1,D10Z1)x2[200],(BCR,ABL1)x2[200],(KMT2Ax2)[200],(ETV6,RUNX1)x2[200]  nuc ish (CRLF2x2)[200],(CDKN2A,D9Z4)x2[200],(JAK2x2)[200] | none |
| **0089** | Bone Marrow: 45,XX,add(6)(q21), dic(9;17)(p12;p11.2)[16]/46,XX[4] | Bone Marrow: nuc ish (D4Z1, D10Z1)x2[200], (BCR, ABL1)x2[200], (KMT2Ax2)[200], (ETV6, RUNX1)x2[200] | Negative for BRAF V600E  Negative for fusions on Hematologic Cancer Fusion Analysis |
| **0092** | Bone Marrow (post treatment): 46,XY[20]  Bone Marrow (pre treatment): N/A- karyotyping not completed on sample | Bone Marrow: nuc ish (D4Z1,D10Z1,BCR,ABL1,KMT2A,ETV6,RUNX1)x2[200](CRLF2,PDGFRB,JAK2)x2[200]  Bone Marrow (pre treatment): N/A- FISH not completed on sample | Positive for CD74::PDGFRB  (targeted B-ALL fusion analysis) |
| **0093** | Bone Marrow: 47,XX,t(1;10)(p21;p13),del(7),(q22q32)[cp1]/47,sl,add(7)(p22),t(9;18)(q22;q21),t(13;19)(q22;q13.1),+mar1[cp4]/46,-47,sdl1,-10 [cp7]/46,XY[8].ish er(18)t(9;18)(ABL1+,D18Z1+),der(19)t(13;19)(LAMP1+) | Bone Marrow: nuc ish (D7Z1x2,D7S486x1)[166/207],(D8Z2x2)[200] | PHF6 (p.H329R)  ASXL1 (p.G646fs*12)  CREBBP deletion exons 7-13  (Foundation One Heme panel) |
| **0096** | Lymph Node: N/A- karyotyping not completed on sample | Lymph Node: N/A- FISH not completed on sample | none |
| **0120** | Forehead Mass: N/A- karyotyping not completed on sample | Forehead Mass: N/A- FISH not completed on sample | none |
| **0194** | Lymph Node: 46,XY,t(8;22)(q24.2;q11.2)[cp19]/ 46,XY[1] | Lymph Node: nuc ish(MYCx2)(5'MYCsep3'MYCx1)[179/200], (BCL6,IGH,BCL2)x2[200] | none |
| **0196** | Muscle Biopsy: N/A-karyotyping not completed on samples | Muscle Biopsy: N/A-FISH not completed on samples | none |
| **0242** | Peripheral Blood: N/A- karyotyping not completed on sample | Peripheral Blood: N/A- FISH not completed on sample | none |
| **0252** | Chest Wall Mass: N/A-no dividing cells | Chest Wall Mass: nuc ish(MECOMx2)[200],(RUNX1T1x2,RUNX1x3)[86/200],(KMT2Ax2)[151],(PML,RARA)x2[200], (CBFBx2)[200], (TP53x2)[200]  nuc ish(D5S23:D5S721,EGR1)x2[200],(D7Z1,D7S486)x2[200],(D8Z2x2)[200] | none |
| **0300** | Lymph Node: N/A- karyotyping not completed on sample | Lymph Node: N/A- FISH not completed on sample | *CIITA::RMI2*  (Hematologic Cancer Fusion Analysis) |
| **0329** | Blood: 46,XY,t(4;11)(q21;q23)[20] | Blood: nuc ish(D4Z1,D10Z1)x2[200],(ABL, BCR)x2[200],(KMT2Ax2)(5’KMT2A sep 3’KMT2Ax1)[170/200],(ETV6,RUNX1)x2[200]  nuc ish (D5S23:D5S721,CSF1R)x2[200],(D7Z1,D7S4B6)x2[200],(D8Z2x2)[200] | *KRAS* (p.G12D)  (Hematologic Neoplasm Mutation Panel) |
| **0371** | Bone Marrow: 57,XY,+X,+5,+6,i(7)(q10),+10,+14,+15,+17,+18,+21,+22 [2, one w/nonclonal]/ 46,XY[18] .ish i(7)(CUL1+,CUX1+,D7Z1+,CUX1,CUL1+) | Bone Marrow: nuc ish (D4Z1x2,D10Z1x3)[108/207], (ABL1x2,BCRx3)[104/200], (KMT2Ax2)[200], (ETV6x2, RUNX1x3)[18/200]/ (ETV6x2,RUNX1x4)[101/200] | none |
| **0397** | Suprasellar Brain Mass: N/A-karyotyping not completed on sample | Suprasellar Brain Mass: N/A-FISH not completed on sample | none |
| **0420** | Neck Mass: N/A-karyotyping not completed on sample | Neck Mass: N/A-FISH not completed on sample | none |
| **0423** | Bone Marrow: 46,XY,t(8;22)(q24.2;q11.2)[10]/46,XY[11] | Bone Marrow: nuc ish(MYCx2)(5'MYC sep 3'MYCx1)[9/200] | none |

**Supplementary Table 5. Prognostic Findings**

| **Patient**  **(IGMCH#)** | **Diagnosis** | **Somatic Variant** | **Type of Variant** | **Individual Variant Impact** | **Predicted Clinical Prognostic Impact** | **Outcome** |
| --- | --- | --- | --- | --- | --- | --- |
| **0009** | ALCL | *NPM**1::ALK* (NM_002520, NM_004304)^52-53, 86-87^ | Fusion | Favorable | Favorable | NED |
| **0056** | T-ALL | *NOTCH1* c.5074T>C(p.Cys1692Arg) (NM_017617.4)^75^  *NOTCH1* c.7375C>T(p.Gln2459Ter) (NM_017617)^75^ | SNV  SNV | Favorable  Favorable | Favorable | NED |
| **0089** | B-ALL | *PAX5* c.239C>G(p.Pro80Arg) (NM_016734)^88-89^  *TP53* c.526T>C(p.Cys176Arg) (NM_000546)^35^  *TP53* loss^35^  *TP53* c.846_847insAGCAGGG(p.Arg283SerfsTer25) (NM_000546)^35^ | SNV  SNV  CNV  Indel | Mixed  Poor  Poor  Poor | Mixed | NED |
| **0093** | AML | *PHF6* c.986A>G(p.His329Arg) (NM_001015877.1)^43, 45-46^  *WT1* c.1319G>A(p.Arg440Lys) (NM_024426.4)^51, 90-92^  *ASXL1* c.1933_1934dupGG(p.Gly646ValfsTer58) (NM_015338.5)^52-53, 86-87^  *CREBBP* deletion exons 7-13 Chr 16 (NM_004380.2)^54^ | SNV  SNV  Indel  Indel | Mixed  Mixed  Poor  Poor | Mixed | Relapse/  Deceased |
| **0096** | ALCL | *NPM**1::ALK* (NM_002520.6, NM_004304.5)^52-53, 86-87^ | Fusion | Favorable | Favorable | NED |
| **0120** | B-ALL | *NT5C2* c.1100G>A(p.Arg367Gln) (NM_012229.4)^93-96^  *KMT2**A::MLLT4*^82^ | SNV  Fusion | Poor  Poor | Poor | Relapse/  Deceased |
| **0242** | B-ALL | *NSD2* c.3448A>G(p.Thr1150Ala) (NM_001042424.3)^64-65^  *TCF**3::PBX1* (NM_003200.5, NM_002585.4)^76^ | SNV  Fusion | Poor  Intermediate | Mixed | Relapse |
| **0252** | AML and  WT | *ASXL1* c.1900_1922delAGAGAGGCGGCCACCACTGCCAT (p.Glu635ArgfsTer15) (NM_015338.6>NM_015338.5)^52-53, 86-87^  *FLT3-*ITD (M_004119.3>NM_004119.2)^83-85^ | Indel  Internal Tandem Duplication | Poor  Poor | Poor | Relapse/  Deceased |
| **0371** | B-ALL | Chromosomes 5, 6, 7q, 10, 14, 17, 18, 21, 22, X gains; 7p loss^54, 77^ | CNV | Favorable | Favorable | NED |

ALCL – anaplastic large cell lymphoma; T-ALL – T-cell acute lymphoblastic leukemia; B-ALL – B-cell acute lymphoblastic leukemia; AML – acute myeloid leukemia; WT – Wilm's Tumor; SNV – single nucleotide variant; indel – insertion/deletion; CNV – copy number variant; NED – no evidence of disease

**Supplementary Table 6. Germline Findings**

| **Patient (IGMCH#)** | **Diagnosis** | **Type** | **Finding** |
| --- | --- | --- | --- |
| **0056** | T-ALL | Carrier Finding | *CFTR* c.1865G>A(p.Gly622Asp) (NM_000492.3)  (Cystic Fibrosis) |
| **0192** | B-ALL | Cancer Predisposition | *PTPN11* c.317A>C (p.Asp106Ala)  (NM_002834.5)  (Noonan Syndrome)* |
| **0194** | Burkitt Lymphoma and  Neuroblastoma | Cancer Predisposition | *CHEK2* c.1100delC (p.Thr367fs)  (NM_007194.3) |
| **0242** | B-ALL | Medically Actionable Finding | 2q21.4-q22.2 loss including *CXCR4*  (WHIM Syndrome) |
| **0252** | AML | Carrier Finding | *GBA* c.680_681delinsGG (p.Asn227Arg)  (NM_000157.4>NM_000157.3)  (Gaucher Disease) |

*previously known

T-ALL – T-cell acute lymphoblastic leukemia; B-ALL – B-cell acute lymphoblastic leukemia; AML – acute myeloid leukemia; SNV – single nucleotide variant; indel – insertion/deletion

**Supplementary Table 7. Patient Summaries**

| **Patient (IGMCH#)** | **Diagnosis** | **Somatic Variants** | **Germline Findings** | **Source of Germline Samples** |
| --- | --- | --- | --- | --- |
| **0009** | ALCL | *NPM**1::ALK* (NM_002520, NM_004304) | None | Blood |
| **0056** | T-ALL | *PHF6* c.309C>A(p.Tyr103Ter) (NM_032458.2)  *JAK1* c.2108G>T(p.Ser703Ile) (NM_001321854.1)  *STAT5B* c.1924A>C(p.Asn642His) (NM_012448.3)  *NOTCH1* c.5074T>C(p.Cys1692Arg) (NM_017617.4)  *NOTCH1* c.7375C>T(p.Gln2459Ter) (NM_017617) | *CFTR* c.1865G>A(p.Gly622Asp) (NM_000492.3) | Buccal |
| **0086** | B-ALL | *PAX5* c.80T>C(p.Phe27Ser) (NM_001280555.1)  *PA*X5 LOH  *CDKN2A* loss  *ZCCHC**7*::*PAX5* (NM_001289119, NM_001280555)  *NRAS* c.183A>T (p.Gln61His) (NM_002524.4) | None | Blood |
| **0089** | B-ALL | *PAX5* c.239C>G(p.Pro80Arg) (NM_016734)  *TP53* c.526T>C(p.Cys176Arg) (NM_000546)  *PAX5* loss  *CDKN2A* loss  *TP53* loss  *TP53* c.846_847insAGCAGGG(p.Arg283SerfsTer25) (NM_000546)  *NRAS* c.35G>A (p.Gly12Asp) (NM_002524) | None | Blood |
| **0092** | B-ALL | *CDKN2A* loss  *CD**74::PDGFRB* | None | Buccal |
| **0093** | AML | *PHF6* c.986A>G(p.His329Arg) (NM_001015877.1)  *BCORL1* c.4258C>T(p.Arg1420Ter) (NM_021946.4)  *WT1* c.1319G>Ap.Arg440Lys (NM_024426.4)  *PICALM::MLLT10* (NM_007166.4, NM_004641.3)  *NF1* c.402_406delTCGGAinsCTTT(p.Arg135PhefsTer30) (NM_000267.3:)  *NF1* c.6925_6926insTCTGGGGGGGC(p.Ser2309PhefsTer14) (NM_001042492.2)  *ASXL1* c.1933_1934dupGG(p.Gly646ValfsTer58) (NM_015338.5)  *CREBBP* deletion exons 7-13 Chr 16 (NM_004380.2) | None | Buccal |
| **0096** | ALCL | *NPM**1*::*ALK* (NM_002520.6, NM_004304.5) | None | Bone Marrow |
| **0120** | B-ALL | *CDKN2A* loss  *KMT2**A*::*MLLT4*  *NT5C2* c.1100G>A(p.Arg367Gln) (NM_012229.4) | None | Buccal |
| **0175** | T-ALL | N/A | None | Buccal |
| **0192** | B-ALL | N/A | *PTPN11* c.317A>C (p.Asp106Ala)  (NM_002834.5) | Blood |
| **0194** | BL and NBL | *DDX3X* c.79C>T(p.Gln27Ter) (NM_001356.4)  *^IGLL5*::*PVT1* (NM_001256296.2, NR_003367.3) | *CHEK2* c.1100delC (p.Thr367fs)  (NM_007194.3) | Blood |
| **0196** | B-ALL | None | None | Blood |
| **0242** | B-ALL | *NSD2* c.3448A>G(p.Thr1150Ala) (NM_001042424.3)  *CDKN2A* loss  Chromosome 6p gain  *TCF**3*::*PBX1* (NM_003200.5, NM_002585.4) | 2q21.4-q22.2 loss including *CXCR4* | Skin Punch Biopsy |
| **0252** | AML and WT | *KRAS* c.37G>T(p.Gly13Cys) (NM_033360.4>NM_033360.3)  *RUNX**1*::*PRDM16* (NM_001754.4, NM_022114.4)  *ASXL1* c.1900_1922delAGAGAGGCGGCCACCACTGCCAT(p.Glu635ArgfsTer15) (NM_015338.6>NM_015338.5)  *FLT3* ITD (M_004119.3>NM_004119.2) | *GBA* c.680_681delinsGG (p.Asn227Arg)  (NM_000157.4>NM_000157.3) | Buccal |
| **0300** | PMBCL | *SOCS1* c.574G>T(p.Ala192Ser) (NM_003745.2)  *SOCS1* c.615C>G(p.Ser205Arg) (NM_003745.2)  *JAK1* c.1872A>T(p.Leu624Phe) (NM_002227.4)  *IL4R* c.127A>C(p.Thr43Pro) (NM_000418.4)  *IL4R* c.598C>T(p.Arg200Trp) (NM_000418.4)  *IRF2BP2* c.295C>T(p.Leu99Phe) (NM_182972.3)  *SOCS1* loss  *B2M* loss  Chromosome 6 loss  Chromosome 9 gain  *CIITA*::*RMI2* (NM_000246.3, NM_152308.3)  *B2M* c.165delT(p.Ile55MetfsTer6) (NM_004048.4) | None | Buccal |
| **0329** | Infant B-ALL | *KRAS* c.35G>A(p.Gly12Asp) (NM_004985.5) | None | Buccal |
| **0371** | B-ALL | Chromosome 7p loss  Chromosomes 5, 6, 7q, 10, 14, 17, 18, 21, 22, X gains | None | Buccal |
| **0397** | AML | None | None | Buccal |
| **0420** | HL and EWS/PNET | None | None | Buccal |
| **0423** | Burkitt Leukemia | *DDX3X* c.1583G>A(p.Arg528His) (NM_001356.5)  *BCL6* c.1760C>A(p.Ala587Asp) (NM_001706.5)  *SMARCA4* c.3574Cc>T(p.Arg1192Cys) (NM_003072.5)  *ID3* c.166C>T(p.Pro56Ser) (NM_002167.5) | None | Buccal |

ALCL – anaplastic large cell lymphoma; T-ALL – T-cell acute lymphoblastic leukemia; B-ALL – B-cell acute lymphoblastic leukemia; AML – acute myeloid leukemia; BL – Burkitt Lymphoma; NBL – Neuroblastoma; WT – Wilm's Tumor; HL – Hodgkin Lymphoma; EWS/PNET – Ewing Sarcoma/Primitive Neuroectodermal Tumor
